# Anti-tumor activity of SL4 against breast cancer cells: induction of G_2_/M arrest through modulation of the MAPK-dependent p21 signaling pathway

**DOI:** 10.1038/srep36486

**Published:** 2016-11-07

**Authors:** Li-Hui Wang, Xiao-Rui Jiang, Guo-Liang Chen, Wei Guo, Jing-Yuan Zhang, Li-Juan Cui, Hua-Huan Li, Meng Li, Xing Liu, Jing-Yu Yang, Chun-Fu Wu

**Affiliations:** 1 Department of Pharmacology, Shenyang Pharmaceutical University, 103 Wenhua Road, 110016 Shenyang, PR China; 2 Key Laboratory of Structure-Based Drugs Design & Discovery of Ministry of Education, Shenyang Pharmaceutical University, 103 Wenhua Road, 110016 Shenyang, PR China

## Abstract

SL4, a chalcone-based compound, has been shown to retard tumor invasion and angiogenesis by suppressing HIF1 activity and to induce apoptosis by promoting ROS release. Here, we report that SL4 is able to inhibit the proliferation of different types of breast cancer cell *in vitro* and *in vivo* by inducing G_2_/M cell cycle arrest. Our results showed that SL4 exhibited strong anti-proliferative activity in several human breast cancer cell lines, with IC_50_ values lower than 1.3 μM. Further studies indicated that SL4 induced G_2_/M arrest in these cell lines. Mechanistically, SL4 reduces the expression of cyclin A2 and cdc25C and decreases the activity of the cdc2/cyclin B1 complex. Notably, SL4 treatment resulted in an obvious increase in p21 mRNA and protein levels through activation of MAPK signaling pathways, but not the TGF-β pathway. SP600125 and PD98059, specific inhibitors of JNK kinase and ERK kinase, significantly blocked the SL4-induced G_2_/M phase arrest and upregulation of p21. Furthermore, SL4 suppressed the growth of established breast tumors in nude mice through upregulation of p21 and downregulation of cdc25C, and displayed a good safety profile. Taken together, these findings demonstrate the potential value of SL4 as a novel multi-target anti-tumor drug candidate.

Breast cancer is the most commonly occurring malignancy and the leading cause of cancer-related death among women world-wide^1^,^2^. Despite earlier diagnosis and development of specific treatments, mortality has only declined by about 30% during the past two decades^1^,^2^. One reason for this is the emergence of drug resistance, which is mainly caused by feedback regulation of single-target agents^3^,^4^; another crucial reason is the absence of specific therapy for triple-negative breast cancers (TNBC), which are characterized by poor prognosis due to a high proliferation rate^5^,^6^. Therefore, the current goal of curing breast cancer may be achieved by finding new therapeutic approaches to reduce the high proliferation of breast cancer cells by targeting multiple intracellular signaling pathways.

The high proliferation characteristics of cancer cells are mainly due to impaired cell cycle regulation^7^,^8^. Therefore, disruption of the cancer cell cycle by therapeutic agents can lead to tumor growth arrest and ultimately to apoptosis, contributing to cancer therapy. Some promising anti-cancer agents which target the cell cycle, such as AZD7762 (Phase I)^9^ and Dinaciclib (Phase II)^10^, are under clinical evaluation. Many more such agents are under preclinical evaluation for cancer treatment, highlighting the promising potential of this strategy in anti-cancer therapy^8^,^11^. Notably, Palbociclib, a small-molecule inhibitor of cyclin-dependent kinase (CDK) 4 and CDK6, has been recently approved in the USA for the first-line treatment of advanced breast cancer^12^, demonstrating the important therapeutic value of small-molecule compounds that target the cell cycle in breast cancer.

Chalcones, which are essential intermediate compounds in flavonoid biosynthesis in plants, have been demonstrated to have anticancer activity in multiple tumor cells^13^,^14^. Our previous study revealed that a novel chalcone-based compound SL4 (also named 5d; Fig. 1A) showed obvious anti-invasive and anti-angiogenic potential by suppressing HIF-1 activity and displayed a remarkable ability to induce cell apoptosis by enhancing ROS accumulation^15^,^16^. Notably, studies by other groups demonstrated that chalcone-based compounds can also arrest the cell cycle in several cancer cells^17^,^18^,^19^. Considering the multi-target potential of chalcone-based compounds, we investigated the anti-tumor effect of SL4 on various different types of breast cancer cell line *in vitro*. Mechanistically, we showed that SL4 induces G_2_/M cell cycle arrest by blocking the activities of the cdc2/cyclin B1 complex and activating the MAPK/p21 pathway. Moreover, we evaluated the *in vivo* anti-tumor activities and safety profiles of SL4 in TNBC tumor mouse models. The results indicated that SL4 may be a potential novel anti-tumor drug candidate and that further investigation is warranted

## Results

### SL4 strongly inhibits the proliferation and viability of human breast cancer cells

**Figure 1:**
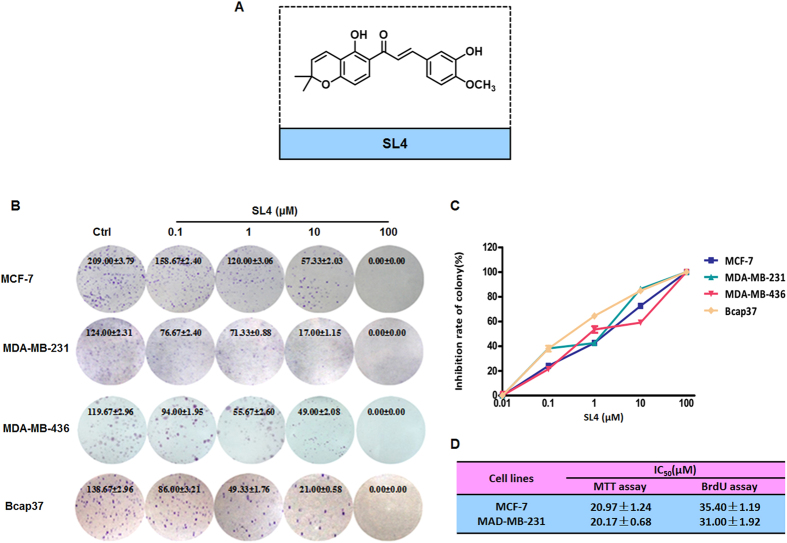
SL4 suppresses proliferation and colony formation of breast cancer cell lines. (**A)** Chemical structure of SL4. (**B**) Effect of SL4 on the colony forming ability of the MCF-7, MDA-MB-231, MDA-MB-436 and Bcap37 cell lines. Cells were incubated with 0.1, 1, 10, and 100 μM SL4 for 24 h. (**C**) Graph showing the concentration-dependent effect of SL4 in the colony formation assay. (**D**) The IC_50_ values detected by MTT and BrdU assay in MCF-7 and MDA-MB-231 cells.

To determine the inhibitory effects of SL4 on breast cancer cell proliferation, we conducted colony formation assays on four human breast cancer cell lines after SL4 treatment. The assays clearly showed that formation of clones by the four tumor cell lines was reduced in a concentration-dependent manner after exposure to SL4 for 24 h (Fig. 1B). The IC_50_ values were 1.1 ± 0.2 μM, 0.5 ± 0.1 μM, 1.3 ± 0.1 μM and 0.3 ± 0.1 μM for MCF-7, MDA-MB-231, MDA-MB-436 and Bcap37 cell lines, respectively. These values are lower than our previously reported IC_50_ (16.9 ± 2.4 μM) for SL4 on normal MCF-10A breast cells^16^. Given their sensitivity to SL4 and their genotype-phenotype characteristics, we selected MCF-7 (ER+, PR+, HER2-, and p53 wild type) and MDA-MB-231 (ER-, PR-, HER2-, and p53 mutation) for subsequent experiments.

Next, we investigated the effects of SL4 on the viability of MCF-7 and MDA-MB-231 cells. MTT assay results showed that SL4 inhibited cell growth with IC_50_ values of 21.0 ± 1.2 μM and 20.2 ± 0.7 μM for the MCF-7 and MDA-MB-231 cell lines, respectively (see Fig. 1D). In addition, the BrdU incorporation assay was performed to confirm the anti-proliferative effects of SL4. The data indicated that the percentages of proliferating MCF-7 and MDA-MB-231 cells were clearly decreased after treatment with various concentrations of SL4 for 24 h, with IC_50_ values of 35.4 ± 1.2 μM and 31.0 ± 1.9 μM for the MCF-7 and MDA-MB-231 cell lines, respectively. Based on the fact that similar IC_50_ values were obtained from both methods, we speculated that the inhibitory action of SL4 on breast cancer cells may be mainly caused by blocking cell proliferation.

### SL4 induces G_2_/M phase arrest of breast cancer cells

**Figure 2:**
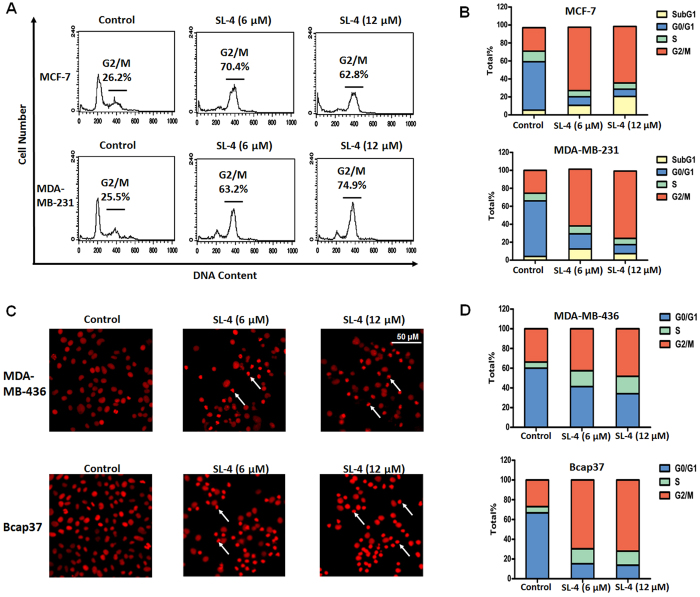
SL4 induces cell cycle arrest in human breast cancer cells. MCF-7, MDA-MB-231, MDA-MB-436 and Bcap37 cells were treated with 6 μM or 12 μM SL4 for 24 h. **(A)** Cell cycle distribution of MCF-7 and MDA-MB-231 was assessed by flow cytometry analysis. (**B**) Quantitation of the percentages of the MCF-7 and MDA-MB-231 cells in each phase of the cell cycle. These experiments were repeated in duplicate. Mean data are presented. (**C**) Cell cycle distribution of MDA-MB-436 and Bcap37 was assessed by a high-content system. Cells were treated with propidium iodide (red) and photographed at a magnification of x400 with an ImageXpress-Micro system. White arrows indicate G2/M phase cells. (**D**) Quantitation of the percentages of the MDA-MB-436 and Bcap37 cells in each phase of the cell cycle. The experiments were repeated in triplicate. Mean data are shown.

To determine whether the SL4-induced inhibition of cell proliferation of the breast cancer cells was associated with cell cycle arrest, we investigated the effect of SL4 on the cell cycle of MCF-7 and MDA-MB-231 cells. Flow cytometry data showed that a 24 h exposure of MCF-7 cells to SL4 resulted in an obvious inhibition of cell cycle progression. There was a marked increase in the G_2_/M fraction, which was accompanied by a decrease in the G_0_/G_1_ and S phase fractions. For the MCF-7 cell line, the percentage of cells in G_2_/M phase increased from 26.2% in controls (treated with DMSO) to 70.4% and 62.8% in cells treated with 6 and 12 μM SL4, respectively (Fig. 2A,B). Similarly, treatment of MDA-MB-231 cells with SL4 for 24 h led to an obvious increase in the number of cells in G_2_/M phase from 25.5% (DMSO control) to 63.2% (6 μM SL4) and 74.9% (12 μM SL4) (Fig. 2A,B).

Cell cycle analysis using a high-content system showed that the proportion of cells with tetraploid DNA was increased in the MDA-MB-436 and Bcap37 lines after treatment with SL4 for 24 h (Fig. 2C). As shown in Fig. 2D, the percentage of MDA-MB-436 and Bcap37 cells in G_2_/M phase was increased from 33.7% and 26.9% to 48.2% and 72.0%, respectively, after treatment with 12 μM SL4. Taken together, the above results indicated that the inhibitory effect of SL4 on the proliferation of breast cancer cells correlated with G_2_/M phase arrest.

Following cell cycle arrest of tumor cells, apoptosis is usually considered as the final event. Thus, we further detected the effects of SL4 on the apoptosis of breast cancer cells. As shown in Supplementary Fig. 1, PARP protein, an apoptosis biomarker, was cleaved after treated with SL4 for 48 h in four breast cancer cell lines, suggesting apoptosis is the final event of SL4 induced cell death.

### SL4 regulates cell-cycle-related proteins in breast cancer cells

**Figure 3:**
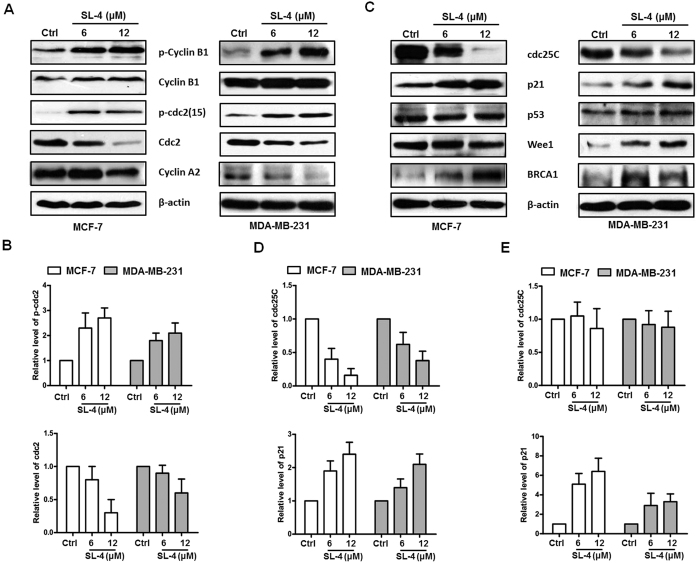
SL4 regulates cell cycle-related proteins in MCF-7 and MDA-MB-231 cells. MCF-7 and MDA-MB-231 cells were treated with SL4 as mentioned in Fig. 2. (**A**) CDK proteins, including Cyclin A2, Cyclin B1, phospho-Cyclin B1, cdc2 and phospho-cdc2(Tyr15), were detected by western blotting. β-actin expression was used as a loading control. (**B**) Protein expression levels of p-cdc2 and cdc2 (relative to β-actin) were determined. Mean ± SD for three replicate determinations. (**C**) CDK-regulated proteins, including p21, p53, Wee1, cdc25C and BRCA1, were detected by western blot. β-actin expression was used as a loading control. (**D**) Protein expression levels of cdc25C and p21 (relative to β-actin) were determined. Mean ± SD for three replicate determinations. (**E**) The mRNA expression levels of *cdc25C* and *p21* were measured by quantitative PCR using specific primers as described in Materials and Methods. *GAPDH* was used as the control. Mean ± SD for three replicate determinations.

The mechanism by which SL4 arrests the cell cycle might involve a direct effect on the expression of cell cycle regulators. Thus, we next investigated the effects of SL4 on cell cycle-related proteins in breast cancer cells. The complex formed by cyclin B1 and cdc2 (also called CDK1) is a specific regulator of G_2_/M phase progression. The complex is activated by the binding of cyclin B1 to cdc2 and phosphorylation of cdc2 at Thr161, and is negatively regulated by the dephosphorylation of cdc2 at Tyr15^7^. We used western blotting to analyze the levels of cyclin B1 and cdc2 in MCF-7 and MDA-MB-231 cells after exposure to SL4. Our results indicated that both cell lines displayed an increase in the protein levels of phosphorylated cyclin B1 and Tyr15-phosphorylated cdc2, and exhibited a concentration-dependent decrease in the protein levels of cdc2, but no alteration in cyclin B1 expression (Fig. 3A,B). Several studies have demonstrated that cyclin A is also involved in the G_2_/M transition, and that the cyclin A/CDK1 complex promotes the activation of the cyclin B1/CDK1 complex^20^,^21^,^22^. Our data showed that cyclin A2 was downregulated by SL4 in both cell lines (Fig. 3B), which is consistent with the arrest in G_2_/M phase.

The activity of cyclin/CDK complexes is regulated by upstream proteins, so we next examined the effect of SL4 on the upstream proteins, including the activator cdc25C, and the inhibitors p21, p53, Wee1 and BRCA1. As shown in Fig. 3C,D, treatment with SL4 for 24 h resulted in a concentration-dependent reduction of cdc25C, and caused an obvious increase in p21 and BRCA1 in both MCF-7 and MDA-MB-231 cells. However, there was no significant change of p53 in either cell line after treatment with SL4. Interestingly, differences were observed in the effect of SL4 on Wee1 expression. The level of Wee1 protein was increased by 24 h exposure to SL4 in MDA-MB-231 cells, but was unaffected in MCF-7 cells, suggesting that the mechanism by which SL4 regulates the cell cycle may be different in the two cell lines.

Given the clear changes induced by SL4 and the important roles of cdc25C and p21 in cell cycle regulation, we also investigated the expression of cdc25C and p21 at the mRNA level. Our data showed that exposure to SL4 for 24 h caused an obvious increase in the p21 mRNA level in both cell lines, while the cdc25C mRNA level was unchanged (Fig. 3E). These results demonstrate that SL4 regulates p21 at the transcriptional level, whereas the regulation of cdc25C may occur at the translational or posttranslational level.

### SL4 affects the TGF-β and MAPK signaling pathways

**Figure 4:**
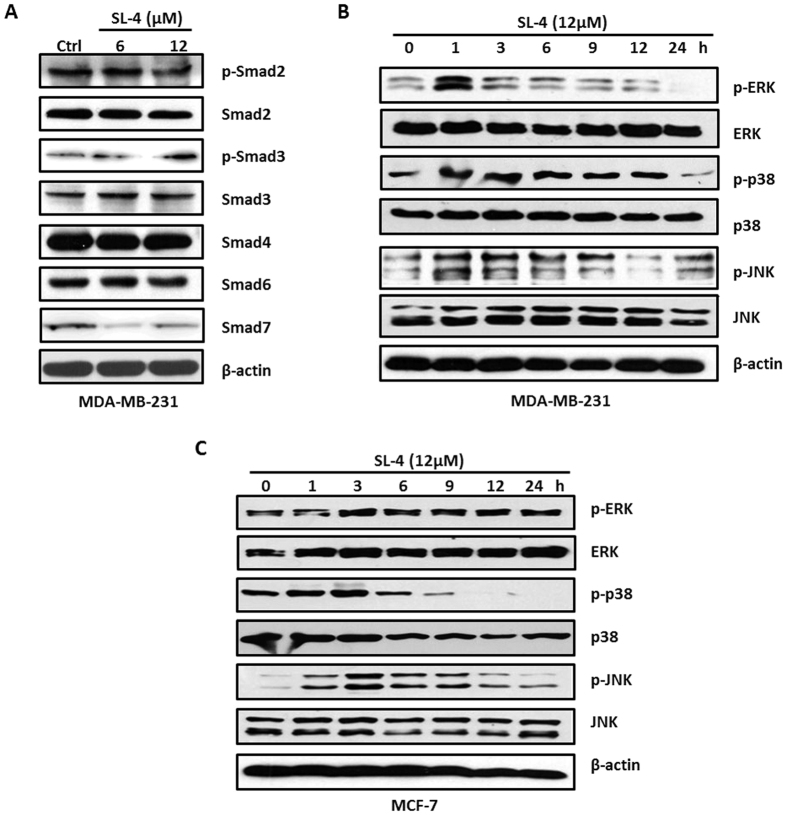
SL4 affects the TGF-β and MAPK pathways. MCF-7 and MDA-MB-231 cells were treated with SL4 at different concentrations or for different lengths of time. (**A**) TGF-β signaling pathway proteins, including Smad2, phospho-Smad2, Smad3, phospho-Smad3, Smad4, Smad6, and Smad7, were detected by western blotting in MDA-MB-231 cells. β-actin expression was used as a loading control. MAPK signaling pathway proteins, including ERK, phospho-ERK, p38, phospho-p38, JNK, and phospho-JNK, were detected by western blot in MDA-MB-231 cells (**B**) and MCF-7 cells **(C)**. β-actin expression was used as a loading control.

The remarkable change of p21 at the mRNA and protein levels prompted us to explore its upstream regulatory pathways. It is well known that the TGF-β signaling pathway can positively regulate p21 expression at the transcriptional level^23^. Thus, we first examined the effect of SL4 on TGF-β signaling using western blotting. As shown in Fig. 4A, after exposure to SL4 for 24 h, MDA-MB-231 cells exhibited an obvious increase in the level of phosphorylated Smad3, and a concentration-dependent decrease in the protein levels of inhibitory Smad7. No significant changes were observed in phosphorylated Smad2, Smad2, Smad4 and inhibitory Smad6. These results indicate that the TGF-β signaling pathway is activated to some extent by SL4 in MDA-MB-231 cells.

Recent evidence has shown that the MAPK signaling pathway also can affect p21 expression at the transcriptional level by co-activating transcription factors of the *p21* gene^24^,^25^. Therefore, we next examined changes in the MAPK signaling pathway. The MAPK pathway is sensitive to the length of the stimulation period, so we analyzed changes in the levels of MAPK proteins in MDA-MB-231 cells after treatment with SL4 for 1–24 h. Our data showed that SL4 treatment for 1 h resulted in an increase in the levels of phosphorylated-ERK, -p38, and -JNK in MDA-MB-231 cells, which suggested an activation of the MAPK/ERK, MAPK/p38 and MAPK/JNK pathways. After treatment with SL4 for 3–6 h, the levels of phosphorylated ERK, p38 and JNK began to decline, and were lower than the levels in untreated cells (0 h) at 24 h (Fig. 4B). A similar pattern was observed in MCF-7 cells (see Fig. 4C). Taken together, our data demonstrate that the regulation of the MAPK signaling pathway by SL4 is time-dependent, involving first activation and then inhibition.

To address whether there is a relationship between HIF-1 pathway and MAPK/ERK pathway, as well as TGF-β pathway, the HIF-1α was silenced by specific siRNA in MDA-MB-231 cells, and the activity of MAPK/ERK pathway and TGF-β pathway were measured by western blot. The results indicated that silence of HIF-1α had no effect on the activation of MAPK/ERK pathway (phosphorylated ERK) and TGF-β pathway (phosphorylated Smad3) triggered by SL4, and also couldn’t affect the SL-4 induced apoptosis in MDA-MB-231 cells (see Supplementary Fig. 2). Therefore, there is no crosstalk between the mechanisms of SL4.

### SL4 induces G_2_/M phase arrest through activation of the MAPK/JNK and MAPK/ERK pathways

**Figure 5:**
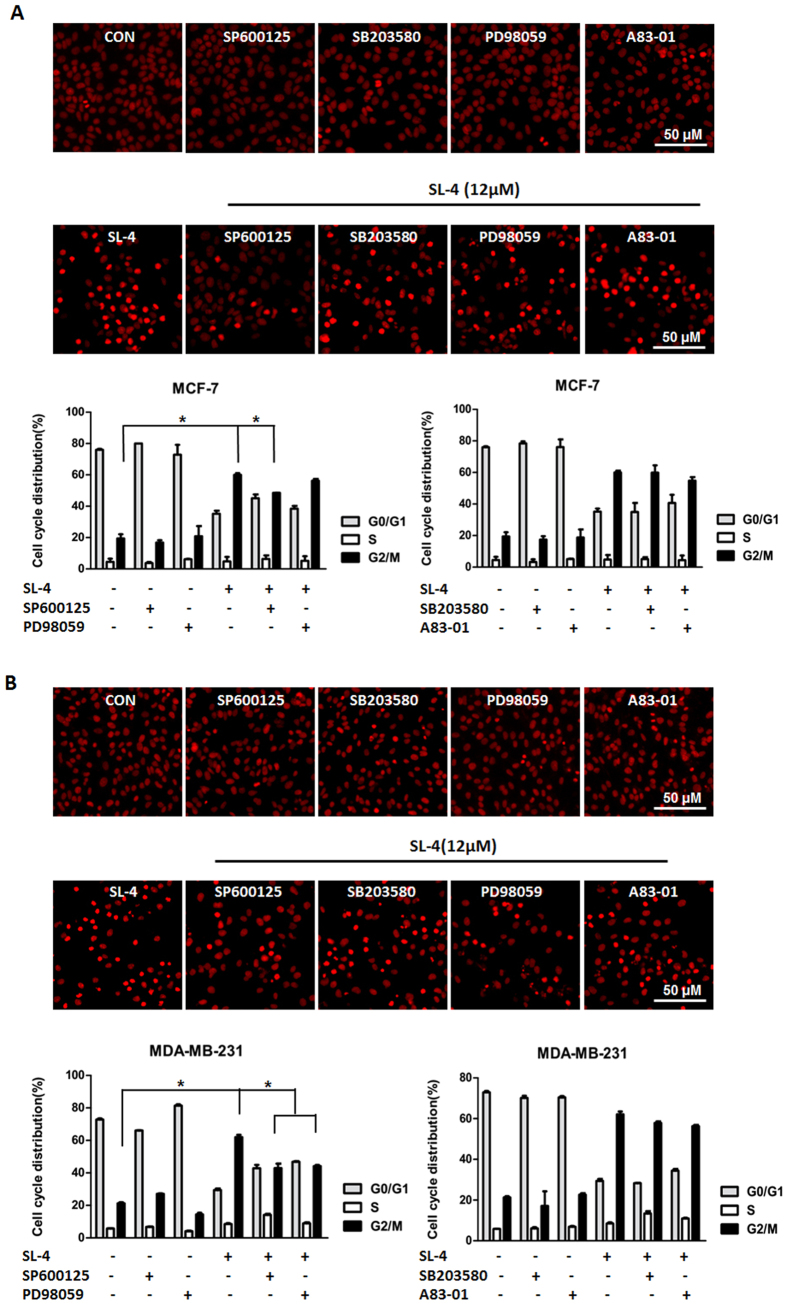
Effect of inhibition of the TGF-β and MAPK pathways on SL4-induced cell cycle arrest. MCF-7 (**A**) or MDA-MB-231(**B**) cells were pre-treated with or without specific inhibitors and then incubated with or without SL4, as indicated. Cell cycle phases were detected by PI staining with a high-content system. The photographs were taken at a magnification of x400 with an ImageXpress-Micro system. Each column in the graph shows the mean for three replicate determinations; bars represent SD. *P < 0.05. (*Denotes that the percentage of cells in G_2_/M phase is significantly higher in the group treated with SL4 alone than in the untreated controls and the groups treated with the indicated specific inhibitors plus SL4).

To explore the role of the MAPK and TGF-β pathways in SL4-induced G_2_/M phase arrest, MCF-7 or MDA-MB-231 cells were pretreated for 30 mins with a specific inhibitor of JNK, SP600125 (10 μM), a specific inhibitor of p38, SB203580 (10 μM), a specific inhibitor of ERK1/2, PD98095 (10 μM), or a specific inhibitor for TGF-β, A83–01 (1 μM). Cells were then exposed to SL4 for 24 h, and cell cycle analysis was performed with a high-content system. Our data showed that compared to the DMSO control, the proportion of G_2_/M phase cells was obviously increased in cultures exposed to 12 μM SL4, and this effect was significantly blocked by the JNK inhibitor SP600125 in both cell lines (see Fig. 5A,B). In contrast, the p38 inhibitor SB203580 and the TGF-β inhibitor A83-01 did not significantly change the proportion of SL4-treated MDA-MB-231 or MCF-7 cells in G_2_/M phase. Interestingly, a clear difference was observed between the two cell lines in the effect of the ERK inhibitor PD98059. Pretreatment with PD98059 significantly reduced the SL4-induced accumulation of G_2_/M phase MDA-MB-231 cells, but had no effect on MCF-7 cells (Fig. 5A,B). This difference may be due to the dissimilar genetic characteristics of the two cell lines. The above data suggest that SL4 induced G_2_/M phase arrest through activation of the MAPK/JNK and MAPK/ERK pathways in breast cancer cells.

### SL4-induced upregulation of p21 is dependent on the activation of MAPK/JNK and MAPK/ERK pathways

**Figure 6:**
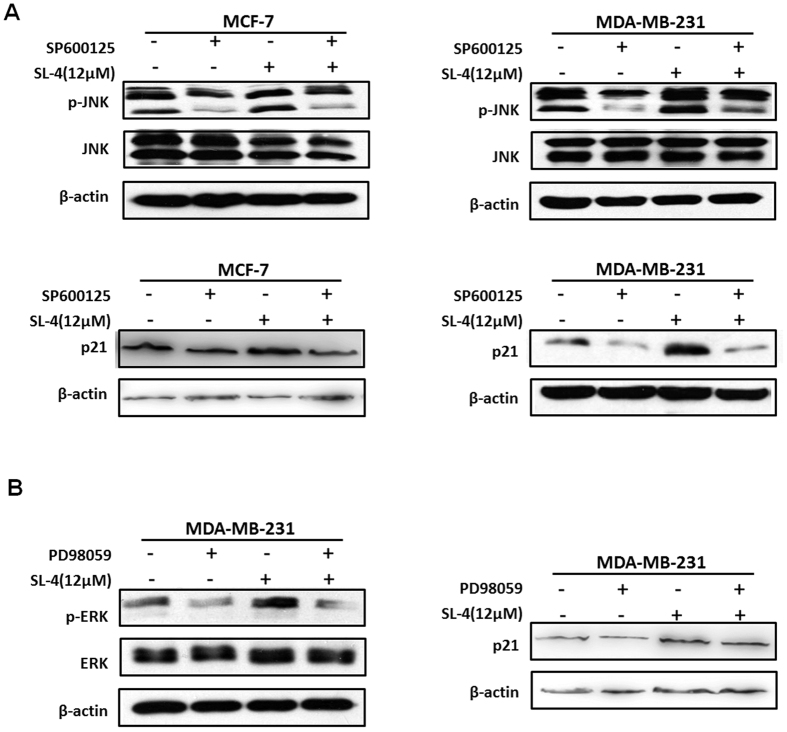
Effect of inhibition of the MAPK pathway on SL4-induced upregulation of p21. (**A**) MCF-7 and MDA-MB-231 cells were pre-treated with or without the specific JNK inhibitor SP600125, then incubated with or without SL4, as indicated. The expression levels of JNK, phosphorylated JNK, and p21 were detected by western blotting. β-actin expression was used as a loading control. (**B**) MDA-MB-231 cells were pre-treated with or without the specific ERK inhibitor PD98059, then incubated with or without SL4, as indicated. The expression levels of ERK, phosphorylated ERK, and p21 were detected by western blot. β-actin expression was used as a loading control.

To further investigate the possible relationship between the p21 and MAPK pathways in SL4-induced cell arrest, we measured the activation status of MAPK and the expression level of p21 in SL4 and/or inhibitor-treated MDA-MB-231 and MCF-7 cells. As expected, exposure of either cancer cell line to JNK inhibitor for 1.5 h resulted in an inhibition of JNK phosphorylation (Fig. 6A). In contrast, the expression of phosphorylated JNK was increased after SL4 treatment for 1 h. Pretreatment with JNK inhibitor led to an inhibition of SL4-induced phosphorylation of JNK. Importantly, the expression level of p21 in both cell lines exhibited a similar pattern to that of phosphorylated JNK after exposure to JNK inhibitor and/or SL4 for 24 h (Fig. 6A). Similarly, in MDA-MB-231 cells exposed to ERK inhibitor and/or SL4, the level of phosphorylated ERK varied in the same way as the level of p21. These data provide further support for an important role of JNK and ERK in activating the target gene p21 and eventually mediating SL4-induced cell cycle arrest.

### SL4 retards tumor growth *in vivo* by regulating cell cycle-related proteins

**Figure 7:**
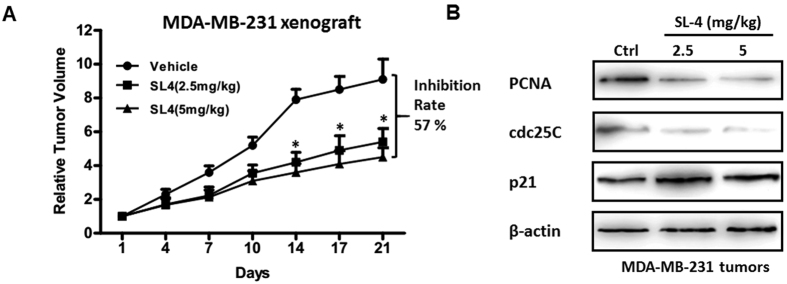
SL4 retards tumor growth in MDA-MB-231 human xenograft mouse models. (**A**) Mice transplanted with MDA-MB-231 human xenografts were randomly divided into three groups and given injections of SL4 (2.5, 5 mg/kg/day, i.v.) or vehicle for a period of three weeks. The relative tumor volumes are expressed as mean ± SD (n = 4–6 per group). All error bars are s.e.m. *Denotes a significant difference (*P* < 0.05) compared to the vehicle control. (**B**) Cell cycle-related proteins, including PCNA, cdc25C, and p21, were analyzed by western blotting in MDA-MB-231 xenograft tumor tissues. β-actin expression was used as a loading control.

To investigate the effect of SL4 on tumor growth *in vivo*, we established an MDA-MB-231 xenograft SCID mouse model. SL4 was administered at a dose of 2.5 or 5.0 mg/kg, in accordance with our previous report^16^. As shown in Fig. 7A, administration of SL4 led to significant dose- and time-dependent inhibitory effects on the growth of MDA-MB-231 tumors when compared with the vehicle control. Administration of SL4 at dosages of 2.5 mg/kg and 5 mg/kg for three weeks resulted in 46% and 57% reduction of the relative tumor volume, respectively. This result suggested that SL4 treatment retarded the growth of MDA-MB-231 xenografts, which was consistent with our observation that SL4 treatment induced cell cycle arrest.

To confirm the mechanism by which SL4 retards tumor growth, we also examined the expression levels of proteins in the xenograft tumors, including the cell cycle proteins p21 and cdc25C, and the cell proliferation marker PCNA. Western blot data showed that SL4 treatment reduced the protein expression of PCNA in MDA-MB-231 xenografts as compared to the control group, suggesting that SL4 retarded tumor growth by inhibiting cell proliferation (Fig. 7B). Furthermore, our data showed that SL4 administration reduced the level of cdc25C protein, while increasing the level of p21 (Fig. 7B), which is consistent with our *in vitro* data. Taken together, our data indicated that SL4 retarded tumor growth *in vivo* by decreasing the expression level of PCNA and cdc25C, and increasing the expression of p21.

### SL4 has a good safety profile *in vivo*

**Figure 8:**
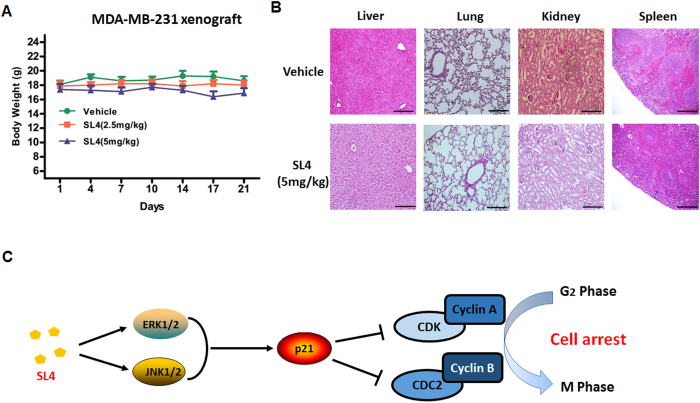
Preliminary safety evaluation of SL4 in SCID mice. Mice transplanted with MDA-MB-231 human xenografts were randomly divided into three groups and given injections of SL4 (2.5, 5 mg/kg/day, i.v.) or vehicle for a period of three weeks. (**A**) The body weights of MDA-MB-231 human xenograft mice after administration of various doses of SL4 or vehicle. (**B**) Histopathological examination of organs from MDA-MB-231 human xenograft mice after administration of SL4 (5 mg/kg) or vehicle. Paraformaldehyde-fixed organs (liver, spleen, lungs and kidneys) were stained by hematoxylin and eosin. Images shown are representatives from both groups. Scale bars represent 100 μm. (**C**) Schematic diagram showing the proposed mechanism by which SL4 causes cell cycle arrest in breast cancer cells.

Our previous study showed that SL4 presented a good therapeutic window because the single maximum tolerated dose (MTD) of SL4 is 200 mg/kg^15^. To further test the potential side effects or toxicity of SL4, we performed a pathological study in SL4-treated MDA-MB-231 xenograft SCID mice. SL4 treatment did not affect the body weight of the mice (Fig. 8A), which means that the dosage used was not overtly toxic. Additionally, histopathological studies indicated that there were no obvious differences in the liver, spleen, lungs and kidneys between the SL4 (5 mg/kg) treated group and the vehicle control group, as judged by microscopic examination of tissue sections (Fig. 8B). These results demonstrated that SL4 displayed a good safety profile even during continuous administration for three weeks.

## Discussion

Accumulating evidence demonstrates that tumor cells display deregulation of multiple cellular signaling pathways^26^. Therefore, treatments with single-target agents usually fail in cancer therapy^26^,^27^. Although combination treatments using distinct single-target agents with chemotherapeutic agents are regarded as more promising, the increased toxicity limits their application. Based on this, the rationale underlying the development of targeting drugs has shifted from focusing on highly specific agents that target single proteins, to a new generation of anti-cancer drugs that affect more than one cancer-related pathway^27^,^28^. We showed in our previous studies that SL4 is a multi-targeting agent which inhibits HIF-1 and activates caspase-dependent pathways^15^,^16^, but nothing is known about its effects on the cell cycle in cancer cells. Here, our results demonstrate that SL4 was able to induce cell cycle arrest at the G_2_/M phase in breast cancer cells by activating the MAPK pathway and subsequently regulating cell cycle-associated proteins, including p21, cdc25C, cdc2, cyclin B1 and cyclin A2. Thus, SL4 may exert anti-tumor activity at different levels: suppressing angiogenesis and metastasis by inhibiting the oncogenic transcription factor HIF, inducing cell apoptosis and arresting the cell cycle.

These three different mechanisms of action of SL4 demonstrate its multi-target potential. In fact, several published studies might provide crucial evidence to elucidate the relationship between these mechanisms. First, cell cycle arrest can induce apoptosis^29^. The final fate of arrested tumor cells, whether arrested at G_0_/G_1_ or G_2_/M, will be apoptosis. Our data showed that SL4 treatment for 48 h resulted in an increase in the level of apoptosis. These results are consistent with the transition model from cell cycle arrest to apoptosis. Additionally, these data also suggest that cell cycle arrest might be the primary anti-tumor mechanism of SL4. Second, HIF might be involved in the regulation of cell cycle arrest. Kilic Eren and his coworkers reported that HIF can negatively regulate the cell cycle kinase inhibitor p21^30^. Moreover, Hubbi *et al*. recently elucidated a transcription-independent mechanism by which the stabilization of HIF-1α leads to cell cycle arrest in response to hypoxia^31^. Additionally, previous reports showed that several HIF-1 inhibitors can induce cell cycle arrest, like SL4^32^,^33^. Taken together, these lines of evidence suggest that there might be an inherent relationship among the three different mechanisms of action of SL4. To address the underlying correlation of SL4 action mechanisms, we studied the relationship between HIF pathway and MAPK pathway, as well as TGF-β pathway, the results indicates that silence of HIF-1α has no effect on the activation of MAPK/ERK pathway and TGF-β pathway triggered by SL4, and also couldn’t affect the SL-4 induced apoptosis. Therefore, SL4 might execute its anti-tumor function through different targets.

Cyclin A2 is a crucial protein that is required for cell cycle transition from S phase to G_2_/M phase^34^. After cells enter into G_2_/M phase, cyclin A2 is replaced by cyclin B. Once cyclin B is activated, cyclin A is no longer needed and is subsequently degraded through the ubiquitin pathway^7^,^11^,^35^. The results in Fig. 2A, showing downregulation of cyclin A2 and upregulation of phosphorylated cyclin B1, indicated that MCF-7 and MDA-MB-231 cells treated with SL4 had completed the transition from G_1_/S phase to G_2_/M phase. Cdc2, as a member of the cyclin-dependent kinase 1 (CDK1) complex with cyclin B1, plays an important role in controlling the transition of cells from G_2_ phase into M phase^7^,^11^. The cyclin B1/cdc2 complex is tightly regulated by the phosphorylation status of cdc2. An activating phosphorylation of cdc2 at Thr161 will prompt cell transition from G_2_ phase into M phase, whereas inhibitory phosphorylation of cdc2 at Thr14 and Tyr15 results in cell cycle arrest in G_2_ phase^36^. Our results revealed that SL4 treatment of MCF-7 and MDA-MB-231 cells resulted in downregulation of cdc2 along with an increase in the level of the inhibitory Tyr15-phosphorylated cdc2, suggesting that regulation of the CDK1 complex is the main mechanism by which SL4 induces G_2_/M phase arrest in breast cancer cells.

Many factors regulate the CDK1 complex^7^,^11^. Among those factors, cdc25C, p21 and Wee1 directly regulate the CDK complex, whereas p53 and BRCA1 indirectly regulate the CDK complex by affecting downstream proteins. Our data showed that SL4 treatment was able to upregulate p21, Wee1 and BRCA1, while also downregulating cdc25C, suggesting that these factors might be involved in the SL4-induced inactivation of the CDK1 complex. Interestingly, consistent with the changes at the protein level, our data also revealed that SL4 treatment contributed to the upregulation of p21 at the mRNA level in breast cancer cells. Considering the crucial role of p21 in cell cycle regulation and the obvious concentration-dependent changes in mRNA and protein levels induced by SL4^23^, p21 might play a dominant role in the regulation of the CDK1 complex by SL4.

The identification of the direct targets of SL4 in cell cycle arrest is important for the further development and applications of SL4 itself and of other chalcone-based compounds. The present results indicated that the effects of SL4 on G_2_/M phase arrest might be mediated via regulation of p21 expression. The TGF-β signaling pathway is considered as a classical upstream pathway for regulating p21 at the transcriptional level^23^, so we investigated whether TGF-β signaling is the target of SL4. Although SL4 treatment activated the TGF-β signaling pathway in MDA-MB-231 cells, inhibition of TGF-β signaling by a specific inhibitor did not affect the SL4-induced G_2_/M phase arrest in breast cancer cells. Therefore, the TGF-β signaling pathway may not be the primary target of SL4. Recent reports showed that the MAPK signaling pathway can also regulate p21 transcription via cooperation with transcription factors^24^,^25^, so we explored the effect of SL4 on MAPK signaling. Our results revealed that treatment with SL4 primarily led to activation of the MAPK/ERK, MAPK/JNK and MAPK/p38 pathways in MCF-7 and MDA-MB-231 cells. Notably, pre-treatment with specific inhibitors of the JNK and ERK pathways, but not the p38 pathway, partially blocked the SL4-induced G_2_/M phase arrest. More importantly, treatment with specific inhibitors of the JNK and ERK pathways not only reduced p21 expression by itself but also reversed the SL4-induced upregulation of p21. Consequently, we suggest that MAPK pathways, especially the ERK and JNK pathways, may be the direct target of SL4 in cell cycle arrest.

In conclusion, this study showed that the multi-target chalcone-based compound SL4 displays favorable efficacy in inhibiting breast cancer, especially TNBC, *in vitro* and *in vivo*. Mechanistically, SL4 induced G_2_/M arrest in breast cancer cells by activating the MAPK/p21 signaling axis, which subsequently regulated the activity of the CDK1/cyclin A2 and CDK1/cyclin B1 complexes (see Fig. 8C). Furthermore, SL4 has shown good safety profiles. Taken together, our results reveal that SL4 is a favorable multi-target small-molecule anticancer drug candidate, and further investigation seems warranted to better understand the relationship between its targets.

## Materials and Methods

### Reagents

SL4 [(E)-1-(5-hydroxy-2,2-dimethyl-2H-chromen-6-yl)-3-(4-trifluoromethylphenyl)-propenone], of purity greater than 98%, was synthesized in the Medicine Chemistry Laboratory at Shenyang Pharmaceutical University (see Fig. 1A). The agent was dissolved in DMSO to 100 mM and stored at −20 °C. MTT (3-(4,5-dimethylthiazol-2-yl)-2,5-diphenyl tetrazolium bromide) was purchased from Sigma, U.S.A. and was dissolved in PBS. Propidium iodide (PI) was purchased from Biosharp and was dissolved in distilled water. SB203580 (a specific inhibitor of MAPK/p38), SP600125 (a specific inhibitor of MAPK/JNK) and PD98059 (a specific inhibitor of MAPK/ERK) were obtained from Sigma Aldrich (St Louis, MO). A83-01, a TGF-β inhibitor, was purchased from Tocris Bioscience (Minneapolis, MN). The primary antibodies against CyclinA2, CyclinB1, phospho-CyclinB1, cdc2, phospho-cdc2(Tyr15), p21, p53, Wee1, cdc25C, BRCA1, PCNA, Smad3, phospho-Smad3, Smad2, ERK1/2, phospho-ERK1/2, p38, phospho-p38, JNK, phospho-JNK, PARP, clv-PARP, and HIF-1α were purchased from Cell Signaling Technology (Danvers, MA); antibodies to β-actin, Smad4, Smad6, Smad7, and specific siRNA for HIF-1α were obtained from Santa Cruz Biotechnology (Santa Cruz, CA).

### Cell lines and cell culture

The human breast cancer cell lines MCF7 (ER+, PR+, HER2-, p53 wild type, BRCA1 wild type), MDA-MB-231 (ER-, PR-, HER2-, p53 mutation, BRCA1 wild type), and MDA-MB-436 (ER-, PR-, BRCA1 mutation) were obtained from the American Type Culture Collection (Manassas, VA). The human breast cancer cell line Bcap37 (ER+) was purchased from the Cell Bank of the Chinese Academy of Sciences (Shanghai, China). They were routinely cultured in Dulbecco’s Modified Eagle’s Medium (high glucose) supplemented with 10% fetal bovine serum (FBS) and maintained at 37 °C in a humidified incubator with 5% CO_2_.

### Colony formation assay

The human breast cancer cell lines were treated with different concentrations of SL4 for 24 h. Then the cells were washed in PBS, seeded in 35 mm dishes and cultured for an additional 2–3 weeks. Finally, the cells were stained with 0.5% crystal violet solution after washing with PBS and fixing with paraformaldehyde, and the colonies (>50 cells) were counted under an inverted microscope. Treatments were carried out in triplicate. The colony formation inhibition rate was calculated as the following formula: Inhibition rate = (1 − various concentrations of colony forming number/control group of colony forming number) * 100%.

### Cell viability assay

The cells (1 × 10^5^ cells/ml) were seeded into 96-well culture plates. After overnight incubation, the cells were treated with various concentrations of SL4 for 24 h. Then 10 μl 3-(4,5-dimethylthiazol-2-yl)-2,5-diphenyl tetrazolium bromide (MTT) solution (2.5 mg/ml in PBS) was added to each well, and the plates were incubated for an additional 4 h at 37 °C. After centrifugation (2500 rpm, 10 min), the medium containing MTT was aspirated, and 100 μl DMSO was added. The optical density of each well was measured at 570 nm with a SpectraMax Paradigm Reader(Molecular Devices).

### BrdU cell proliferation assay

The BrdU Cell Proliferation Assay Kit detects 5-bromo-2′-deoxyuridine (BrdU) incorporated into cellular DNA during cell proliferation using an anti-BrdU antibody. Briefly, cells growing in 96-well plates (5000 cells/well) were treated with different concentrations of SL4 for 24 h, and were then assayed using the BrdU Cell Proliferation Assay Kit (CST, Danvers, MA) according to the manufacturer’s instructions. Each assay was replicated 3 times.

### Flow cytometry analysis

Flow cytometry analysis was performed as previously described^37^. Briefly, about 1–5 × 10^6^ breast cancer cells were harvested at room temperature after pre-treatment with various concentrations of SL4 for 24 h. The supernatant was removed, and the cells were trypsinized, then ice-cold 70% ethanol was added. Ethanol-fixed cells were resuspended in PBS containing 0.1 mg/ml RNase and incubated at 37 °C for 30 min. The pelleted cells were suspended in 1.0 ml of 40 μg/ml propidium iodide (PI) and analyzed using a flow cytometer (Becton Dickinson). The cell cycle distribution was estimated according to standard procedures. The percentage of cells in the different cell cycle phases (G_0_/G_1_, S, or G_2_/M phase) was calculated using CELLQuest (Becton Dickinson) software.

### Cell cycle analysis with a high-content system

High-content analysis of the cell cycle was conducted according to a previous report with minor modification^38^. Briefly, the number of cells and the percentage of cells in different cell cycle phases were assessed in breast cancer cells. The cells were plated at 5 × 10^3^ cells per well in 96-well microplates. After 24 h, the cells were treated with various concentrations of SL4 for 24 h, and then fixed with 70% ethanol overnight. After 2 washes in PBS, cells were stained by PI for 30 mins. Plates were scanned with the ImageXpress Micro (Molecular Devices) automated epifluorescent microscope. For cell number and cell-cycle analysis, the PI integrated intensity was assessed using the cell-cycle application module (MetaXpress). The percentage of PI-positive cells was calculated using the multi-wavelength cell scoring application module (MetaXpress).

### Western blot analysis

About 1 × 10^7^ breast cancer cells were gathered after pre-treatment with SL4 for 24 h or 48 h. Western blotting was performed as previously described^39^. In brief, equal amounts of total protein extracts from cultured cells or tissues were fractionated by 10–15% SDS-PAGE and electrically transferred onto polyvinylidene difluoride (PVDF) membranes. Mouse or rabbit primary antibodies and horseradish peroxidase (HRP)-conjugated appropriate secondary antibodies were used to detect the designated proteins. The bound secondary antibodies on the PVDF membrane were reacted with ECL detection reagents (Thermo Scientific) and exposed in a dark room. Results were normalized to the internal control β-actin.

### Quantitative PCR analysis

Total RNA was isolated from breast cancer cells using an RNeasy Mini Kit (Qiagen) as described in the product insert. The RNA was reverse transcribed with a RevertAid First Strand cDNA Synthesis Kit (Thermo) and PCR was performed using iQ SYBR Green Supermix and a CFX96 Real-Time PCR Detection System (Bio-Rad). Primers used were as follows: glyceraldehyde-3-phosphate dehydrogenase (*GAPDH*) reverse primer 5′-CCC TCA ACG ACC ACT TTG TCA-3′ and forward primer 5′-TTC CTC TTG TGC TCT TGC TGG-3′; *p21*^*WAF-1*^ reverse primer 5′- GTC CAG CGA CCT TCC TCA TCCA-3′ and forward primer 5′-CCA TAG CCT CTA CTG CCA CCA TC-3′; *Cdc25C* reverse primer 5′-TGG AAC TTC CCC GAC AGT AAG G-3′ and forward primer 5′-TTT TTC CAA GGT ATG TGC GCT G-3′.

### *In vivo* anti-tumor efficacy studies

For tumorigenesis assessment, viable MDA-MB-231 cells (5 × 10^6^/100 μl PBS per mouse), as confirmed by trypan blue staining, were subcutaneously injected into the right flank of 7- to 8- week old female SCID mice. When the average tumor volume reached 100 mm^3^, mice were randomly divided into various treatment and control groups (4–6 mice per group). Body weights were recorded once every two days. After about two weeks, mice were sacrificed and the tumors were excised and stored at −80 °C until analyzed by western blotting. At the same time, some internal organs (liver, spleen, lung and kidney) were also collected for pathological study. The *in vivo* experiments were performed in accordance with relevant guidelines and regulations approved by the Committee on the Ethics of Animal Experiments of the Shenyang Pharmaceutical University.

### Histopathological analysis

The excised tissues were fixed in 4% neutral-buffered formalin solution for more than 24 h and embedded in paraffin. Sections of the tissues (3–5 μm) were stained with hematoxylin and eosin.

### Statistical analysis

Statistical analysis was performed using the SPSS11.5 software package for Windows (SPSS, Chicago, IL). Data are presented as the mean ± s.e.m. Statistical significance was calculated using Student’s t-test, with a probability level of *P* < 0.05 considered to be statistically significant.

## Additional Information

**How to cite this article**: Wang, L.-H. *et al*. Anti-tumor activity of SL4 against breast cancer cells: induction of G_2_/M arrest through modulation of the MAPK-dependent p21 signaling pathway. *Sci. Rep.* **6**, 36486; doi: 10.1038/srep36486 (2016).

**Publisher’s note**: Springer Nature remains neutral with regard to jurisdictional claims in published maps and institutional affiliations.

## Supplementary Material

Supplementary Information
