# Supplementary material for: Anti-tumor activity of SL4 against breast cancer cells: induction of G2/M arrest through modulation of the MAPK-dependent p21 signaling pathway
Source: Sci Rep. 2016 Nov 7;6:36486. doi: 10.1038/srep36486 (PMC5098232; doi:10.1038/srep36486)
Supplement: Supplementary Information [file srep36486-s1.pdf]

# Anti-tumor activity of SL4 against breast cancer cells: induction of $G_2$/M arrest through modulation of the MAPK-dependent p21 signaling pathway

Li-Hui Wang[1], Xiao-Rui Jiang[1], Guo-Liang Chen[2], Wei Guo[1], Jing-Yuan Zhang[1], Li-Juan Cui[1], Hua-Huan Li[1], Meng Li[1], Xing Liu[1], Jing-Yu Yang[1*], Chun-Fu Wu[1*]

[1]Department of Pharmacology; [2]Key Laboratory of Structure-Based Drugs Design & Discovery of Ministry of Education, Shenyang Pharmaceutical University, 103 Wenhua Road, 110016 Shenyang, PR China

***Corresponding author：** Department of Pharmacology, Shenyang Pharmaceutical University, Shenyang, China, Tel: 86-24-23986011; Fax: 86-24-23986339; e-mail: wucf@syphu.edu.cn(CF Wu), yangjingyu2011@163.com(JY Yang)

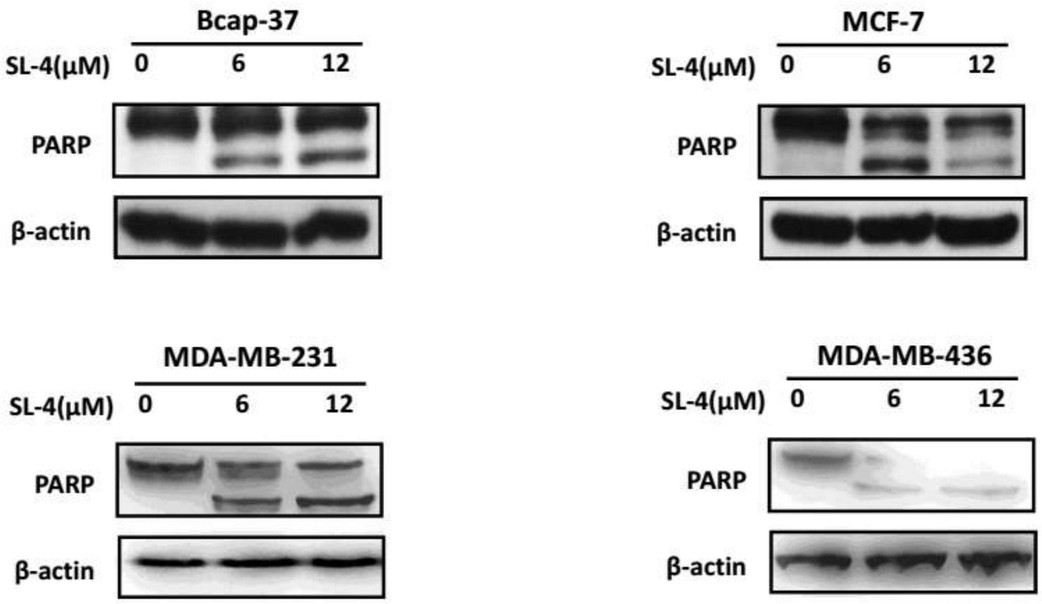


**Supplementary Figure 1. SL4 induces cell apoptosis in breast cancer cell lines.** The cells were treated with SL4 (6 or 12 μM) for 48 h, then the protein was collected. The expression of PARP were detected by western blot. β-actin was used as loading control.

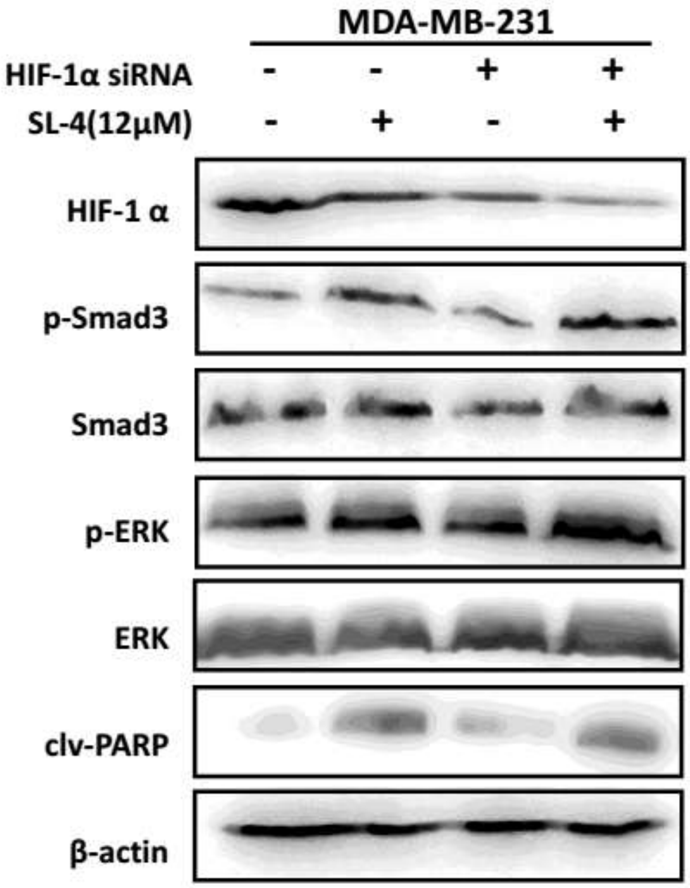


**Supplementary Figure 2. The effects of silence of HIF-1α on MAPK/ERK, TGF-β, and apoptosis induced by SL-4 in MDA-MB-231 cell lines.** The cells were pretreated with HIF-1α siRNA (20 nM) and the same amount of scramble siRNA for 24 h, then exposed to SL4 for 24 h. The expression of HIF-1α, phosphorylated Smad3, Smad3, phosphorylated ERK, ERK, and clv-PARP were detected by western blot. β-actin was used as loading control.

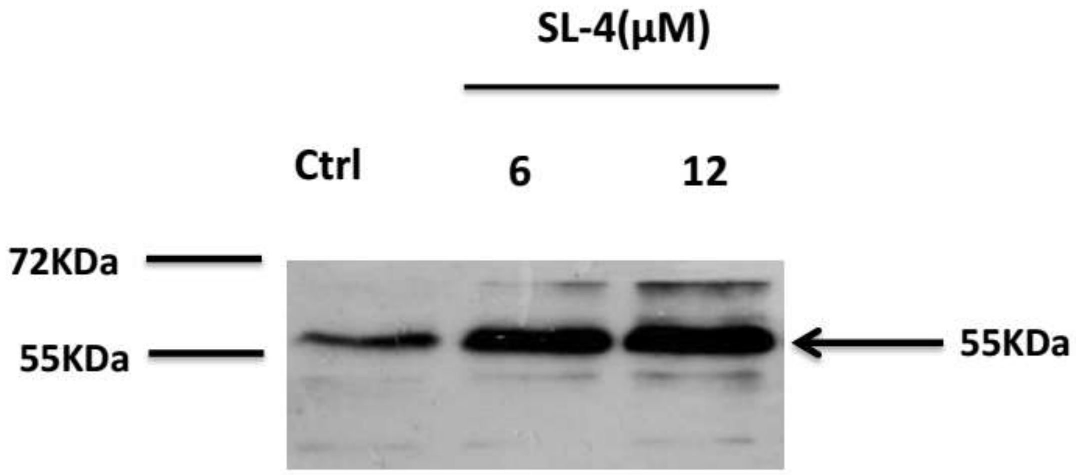


**Supplementary Figure 3-1. The effects of SL-4 on p-CyclinB1 in MCF-7 cells.**

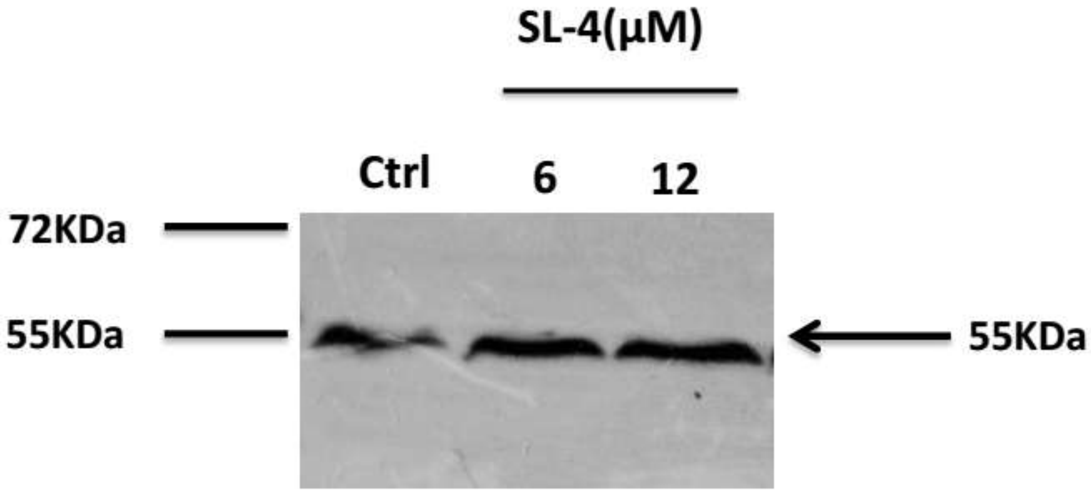


**Supplementary Figure 3-2. The effects of SL-4 on CyclinB1 in MCF-7 cells.**

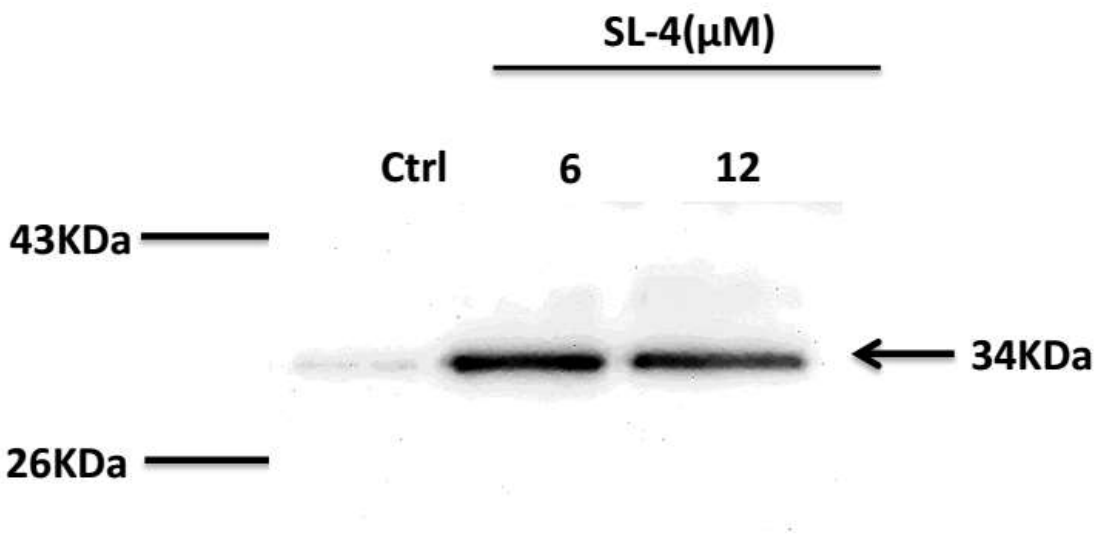


**Supplementary Figure 3-3. The effects of SL-4 on p-cdc2(15) in MCF-7 cells.**

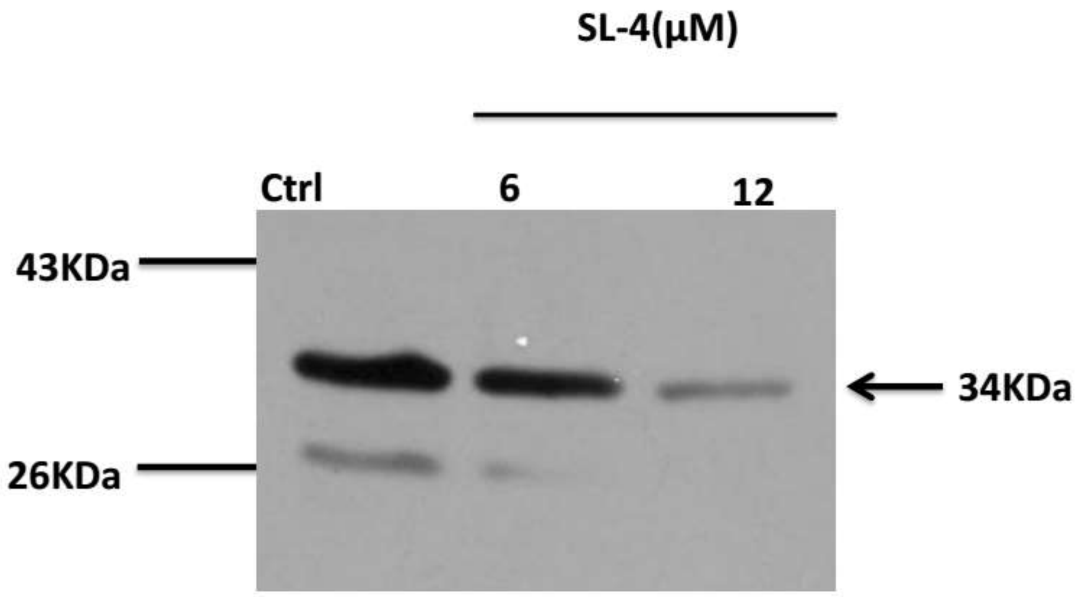


**Supplementary Figure 3-4. The effects of SL-4 on cdc2 in MCF-7 cells.**

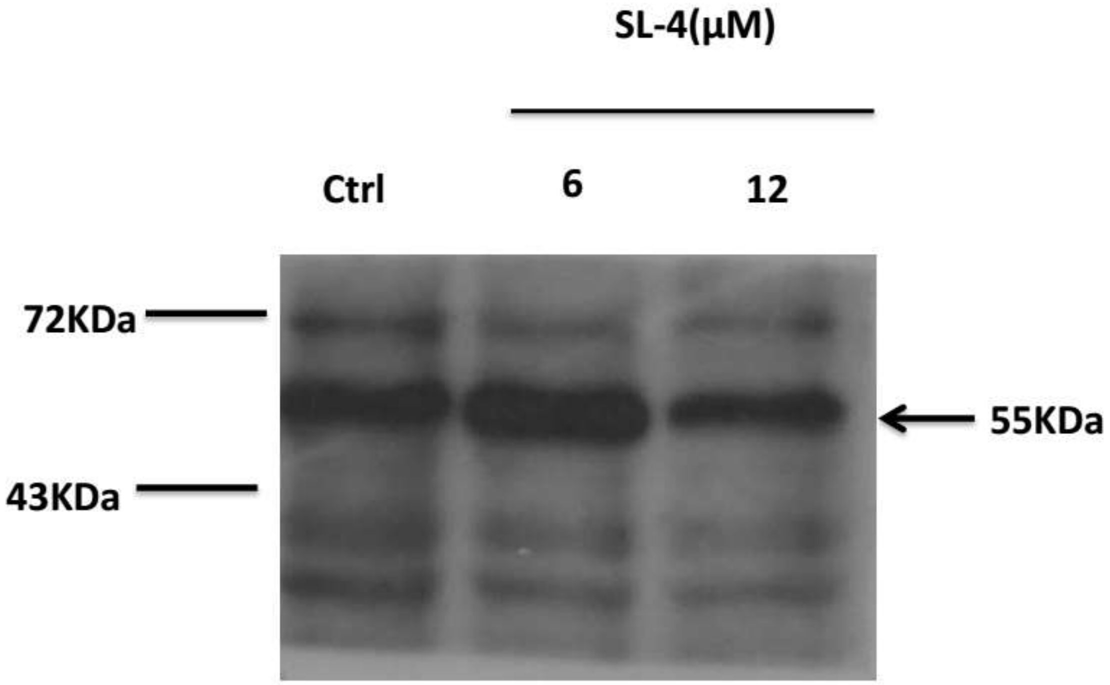


**Supplementary Figure 3-5. The effects of SL-4 on Cyclin A2 in MCF-7 cells.**

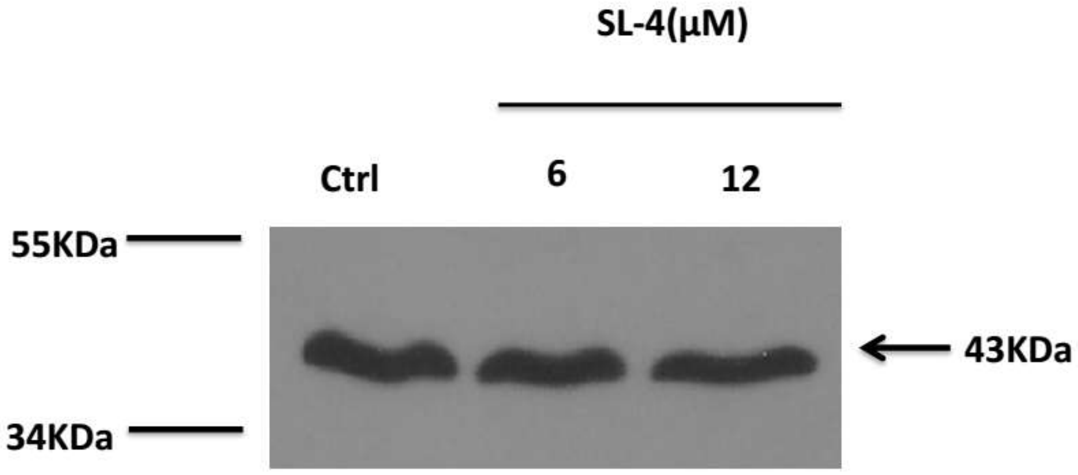


**Supplementary Figure 3-6. Loading control β-actin in MCF-7 cells.**

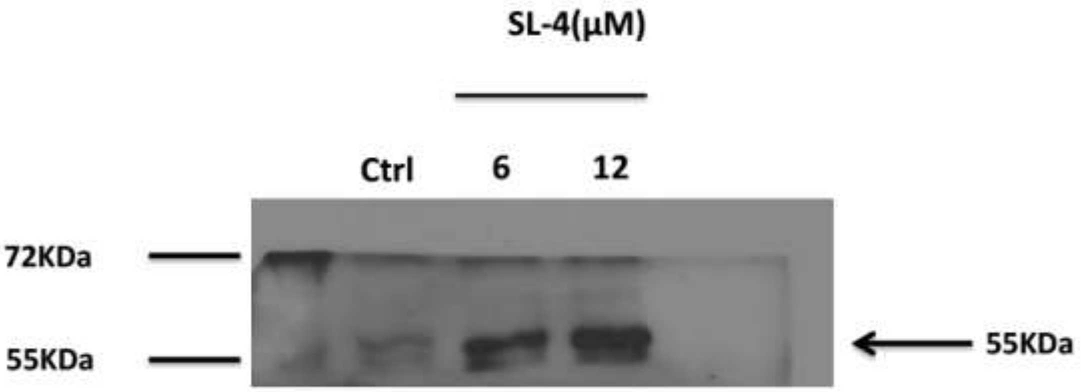


Supplementary Figure 3-7. The effects of SL-4 on p-CyclinB1 in MDA-MB-231 cells.

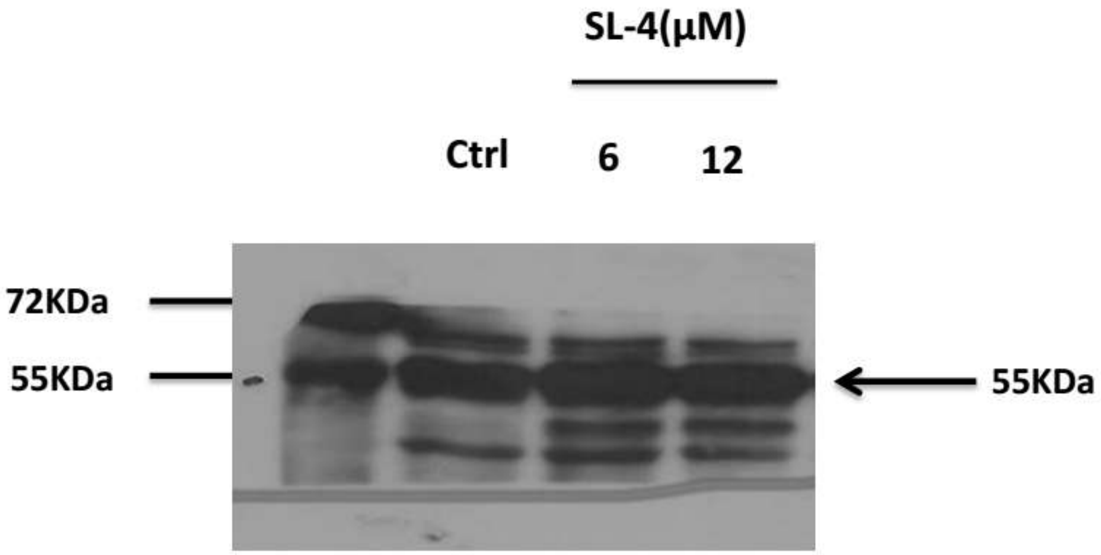


Supplementary Figure 3-8. The effects of SL-4 on CyclinB1 in MDA-MB-231 cells.

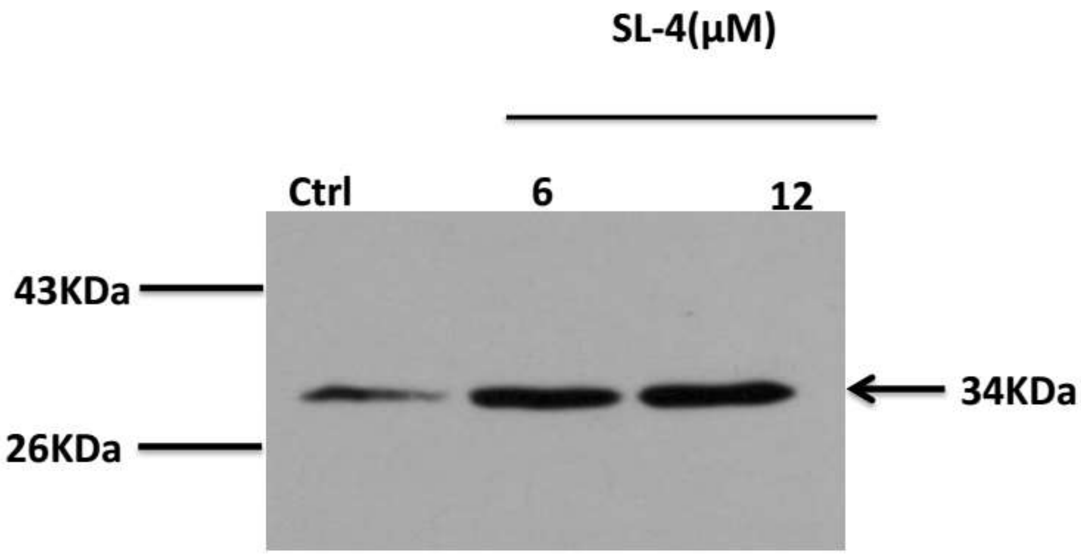


**Supplementary Figure 3-9. The effects of SL-4 on p-cdc2(15) in MDA-MB-231 cells.**

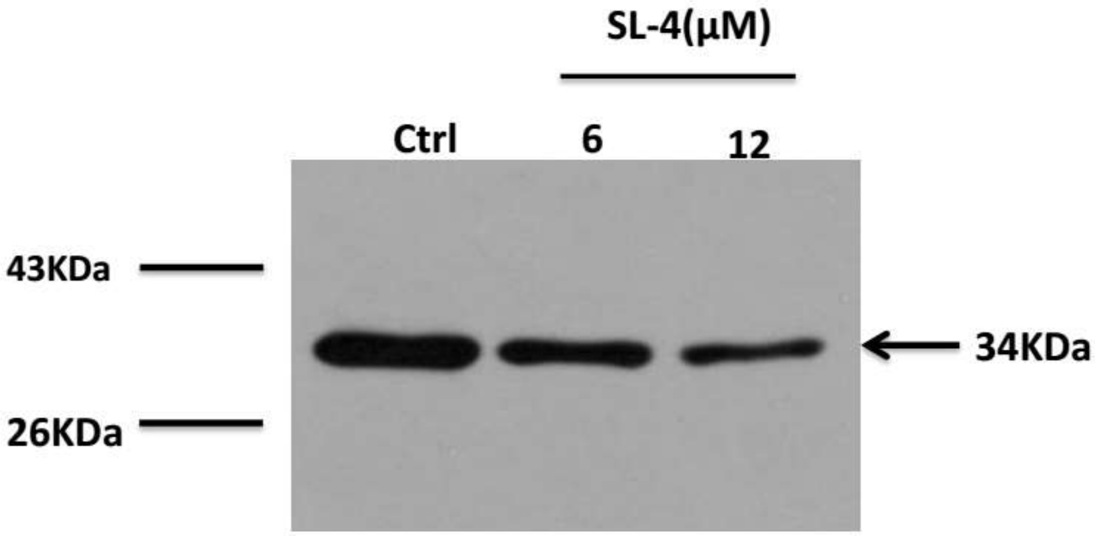


**Supplementary Figure 3-10. The effects of SL-4 on cdc2 in MDA-MB-231 cells.**

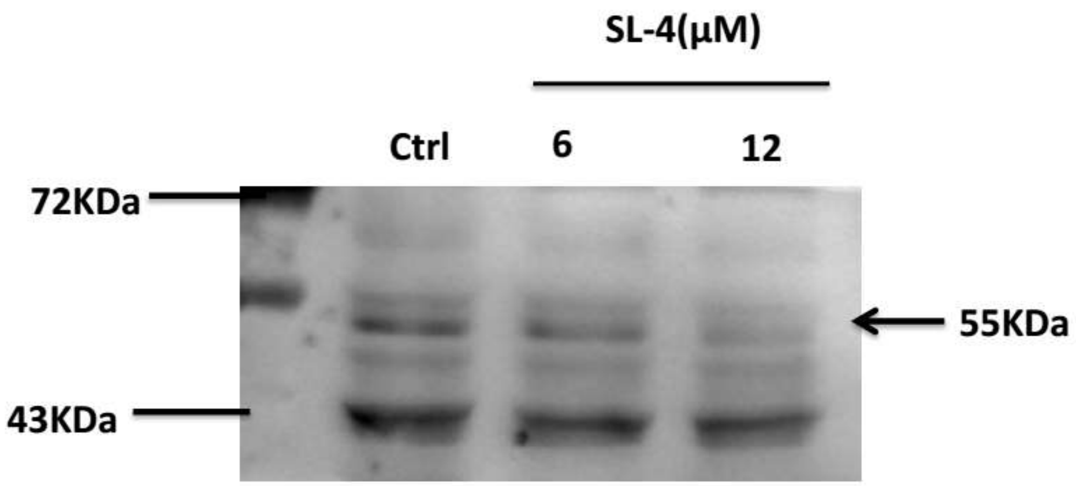


**Supplementary Figure 3-11. The effects of SL-4 on Cyclin A2 in MDA-MB-231 cells.**

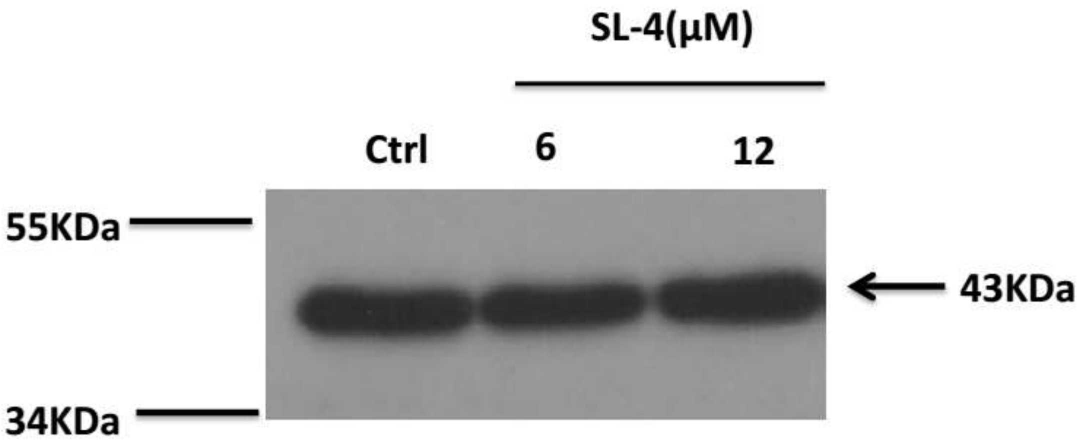


**Supplementary Figure 3-12. Loading control β-actin in MDA-MB-231 cells.**

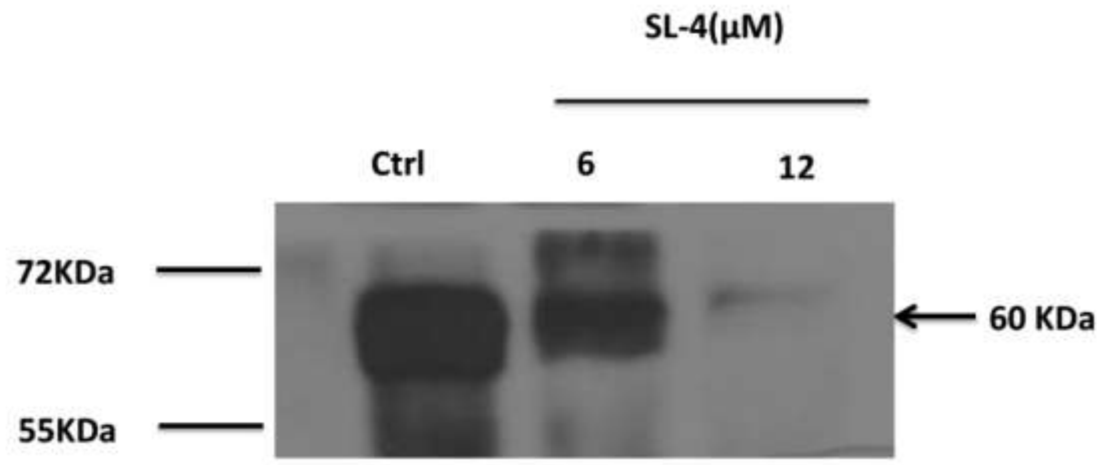


**Supplementary Figure 3-13. The effects of SL-4 on cdc25C in MCF-7 cells.**

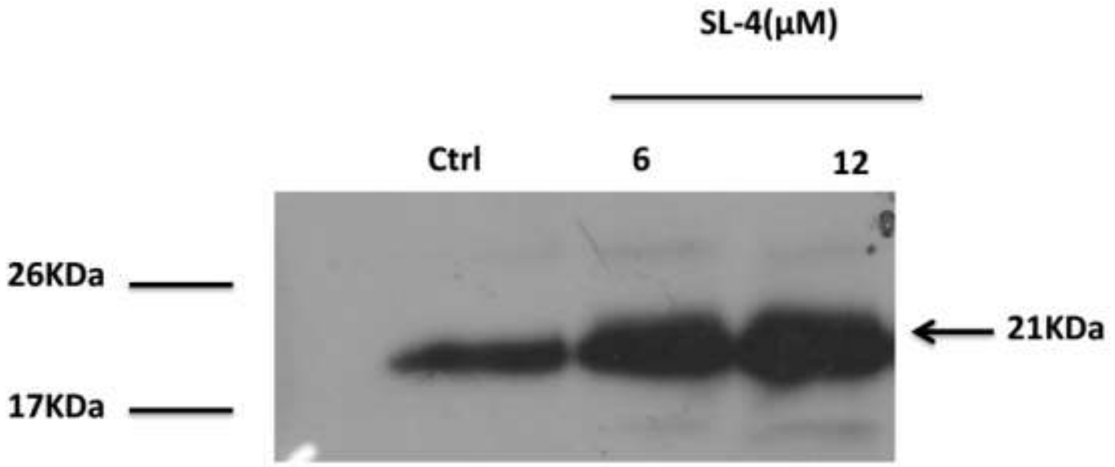


**Supplementary Figure 3-14. The effects of SL-4 on p21 in MCF-7 cells.**

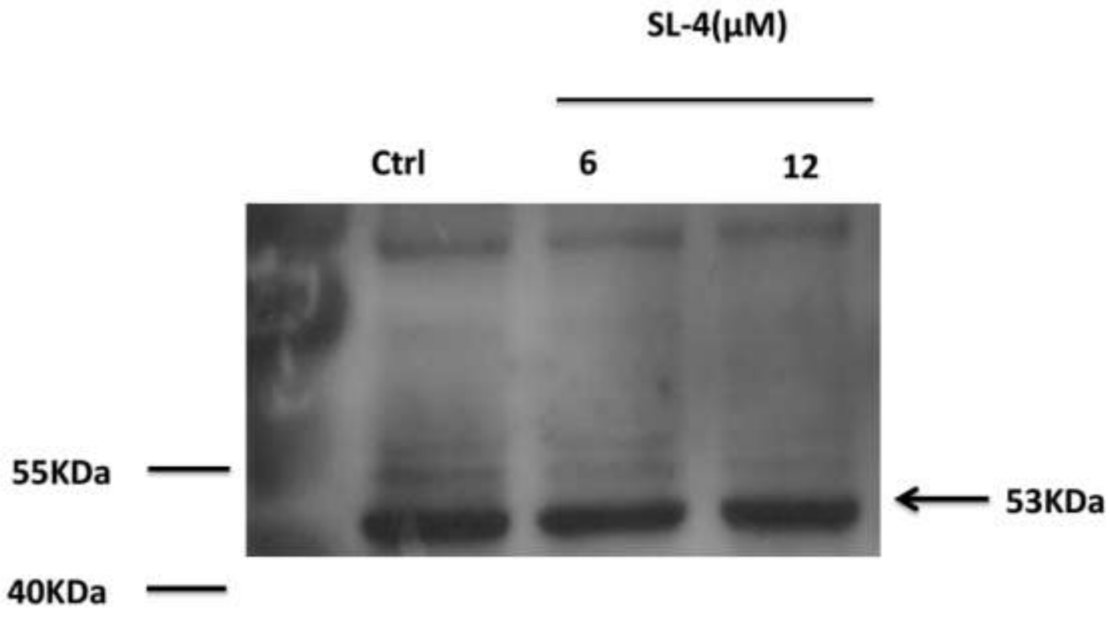


**Supplementary Figure 3-15. The effects of SL-4 on p53 in MCF-7 cells.**

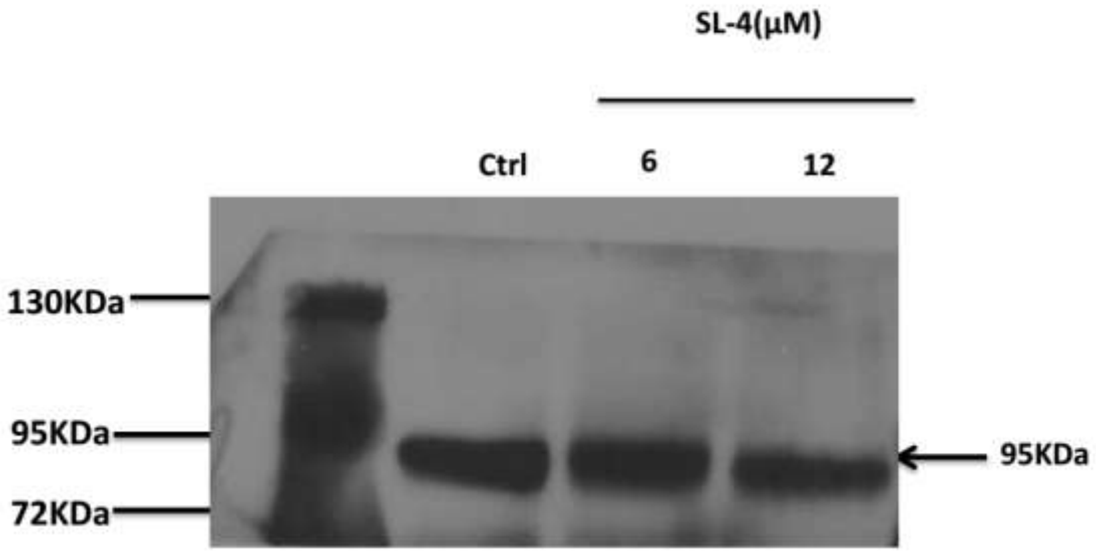


**Supplementary Figure 3-16. The effects of SL-4 on Wee1 in MCF-7 cells.**

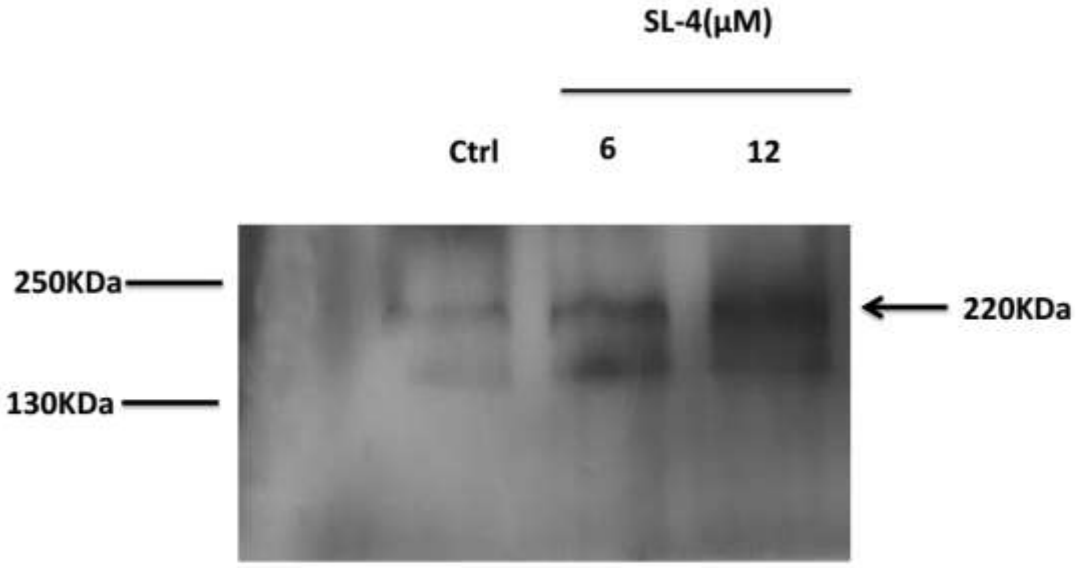


Supplementary Figure 3-17.  The effects of SL-4 on BRCA1 in MCF-7 cells.

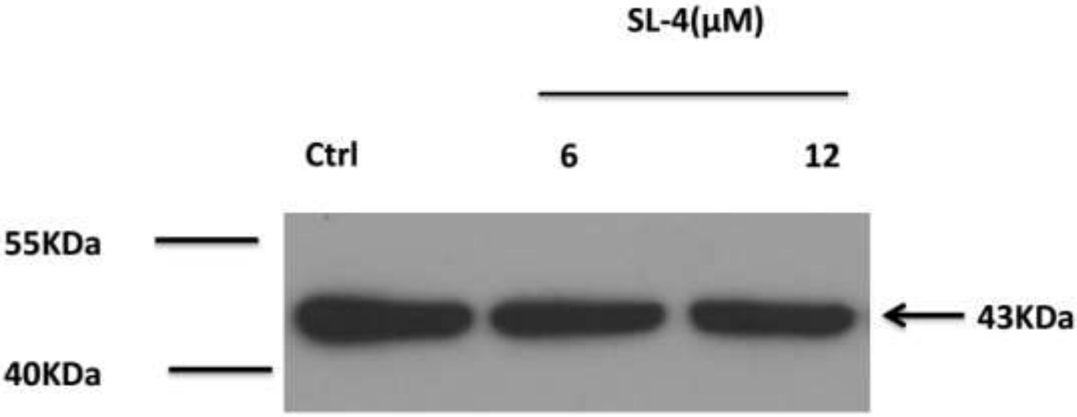


Supplementary Figure 3-18.  Loading control β-actin in MCF-7 cells.

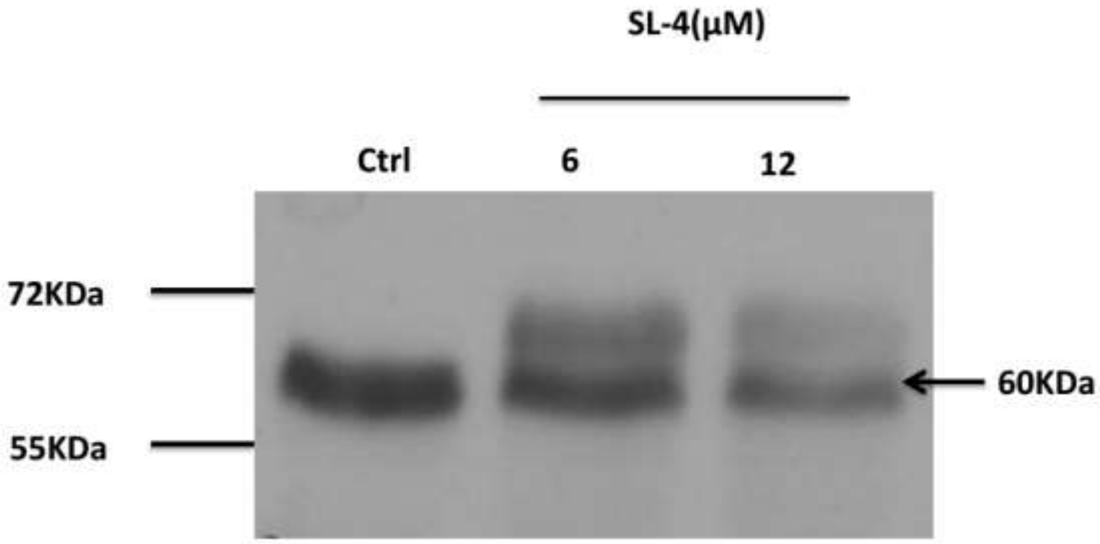


Supplementary Figure 3-19. The effects of SL-4 on cdc25C in MDA-MB-231 cells.

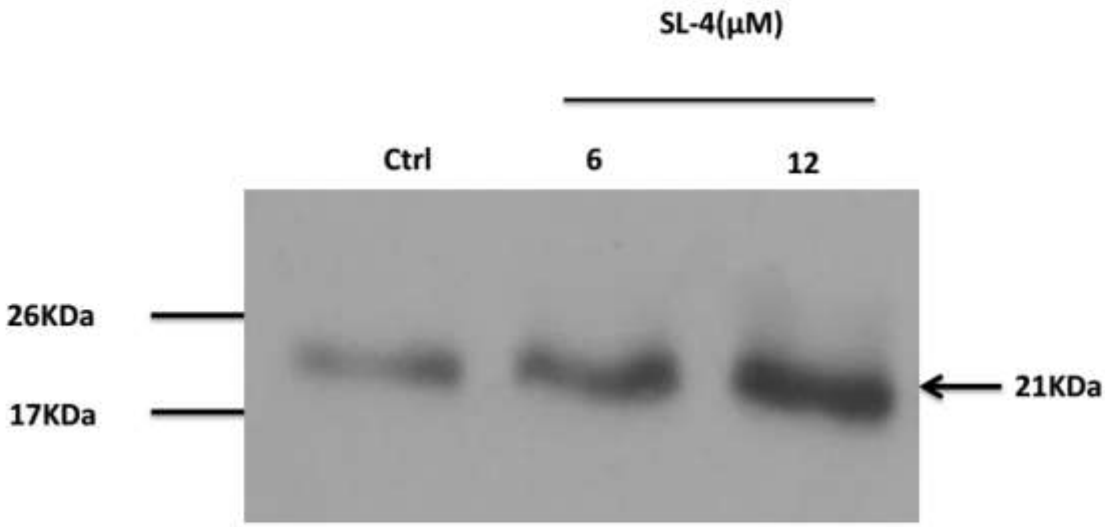


Supplementary Figure 3-20. The effects of SL-4 on p21 in MDA-MB-231 cells.

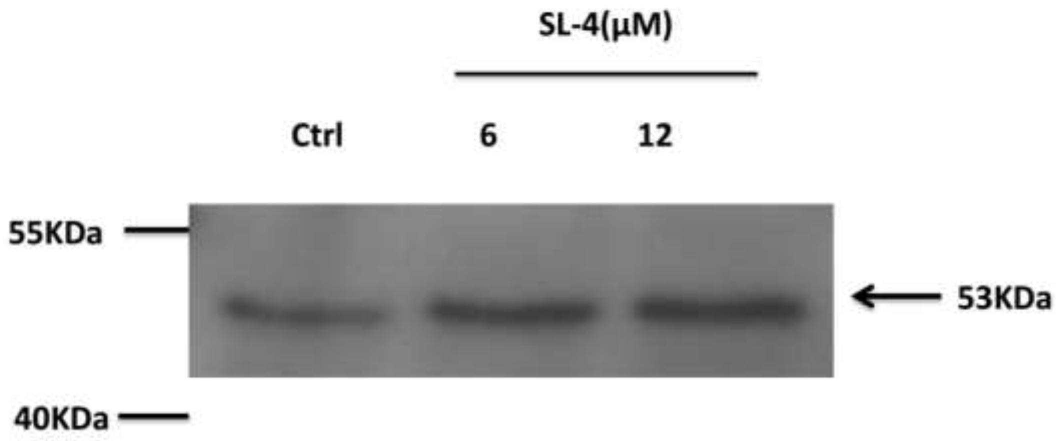


**Supplementary Figure 3-21. The effects of SL-4 on p53 in MDA-MB-231 cells.**

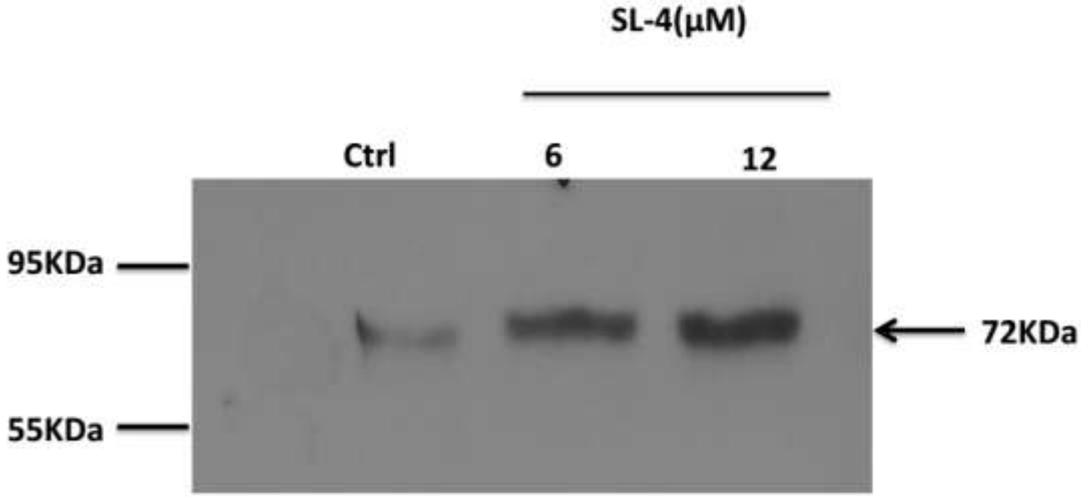


**Supplementary Figure 3-22. The effects of SL-4 on Wee1 in MDA-MB-231 cells.**

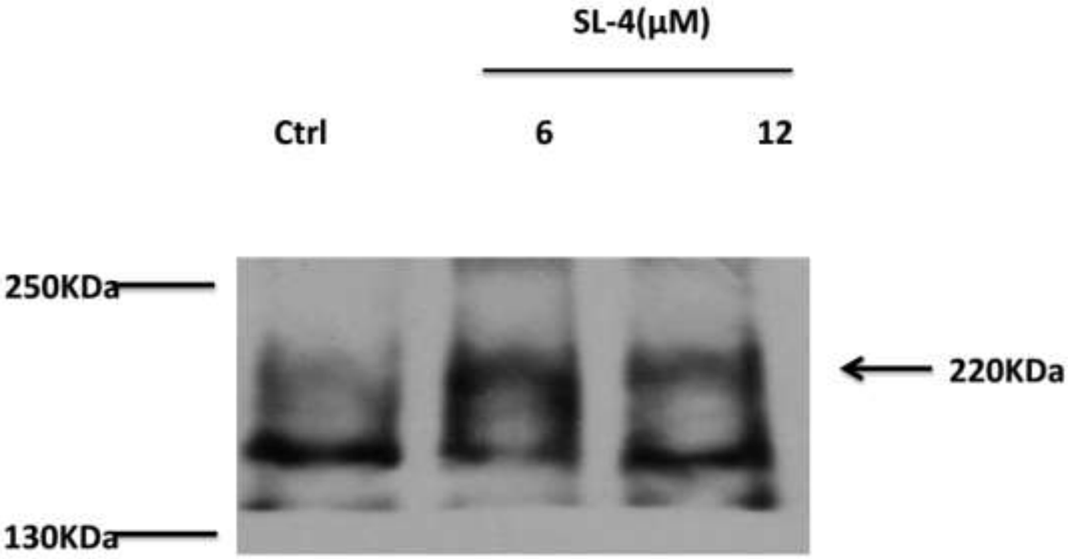


**Supplementary Figure 3-23. The effects of SL-4 on BRCA1 in MDA-MB-231 cells.**

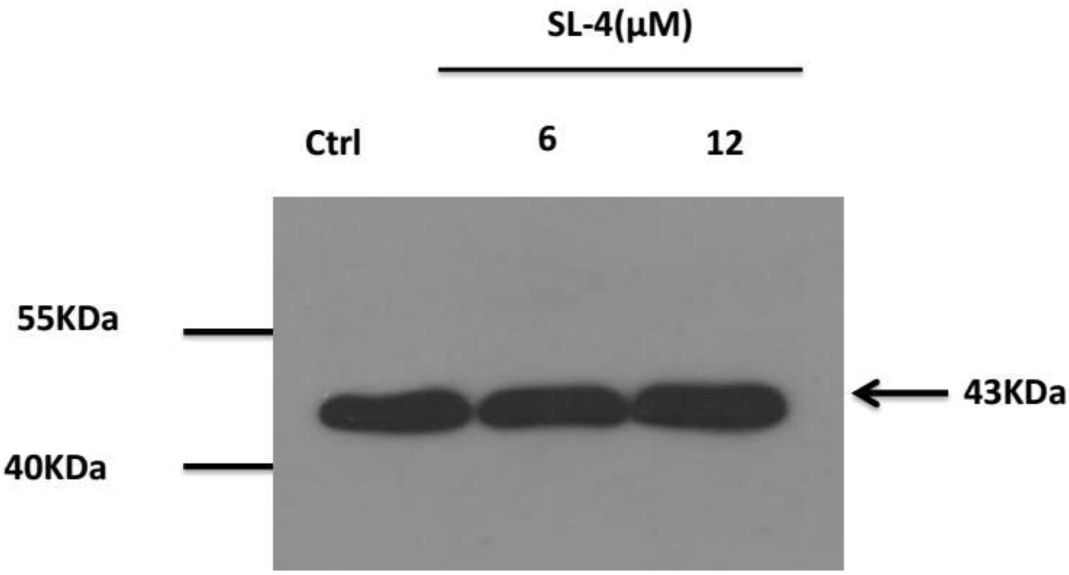


**Supplementary Figure 3-24. Loading control β-actin in MDA-MB-231 cells.**

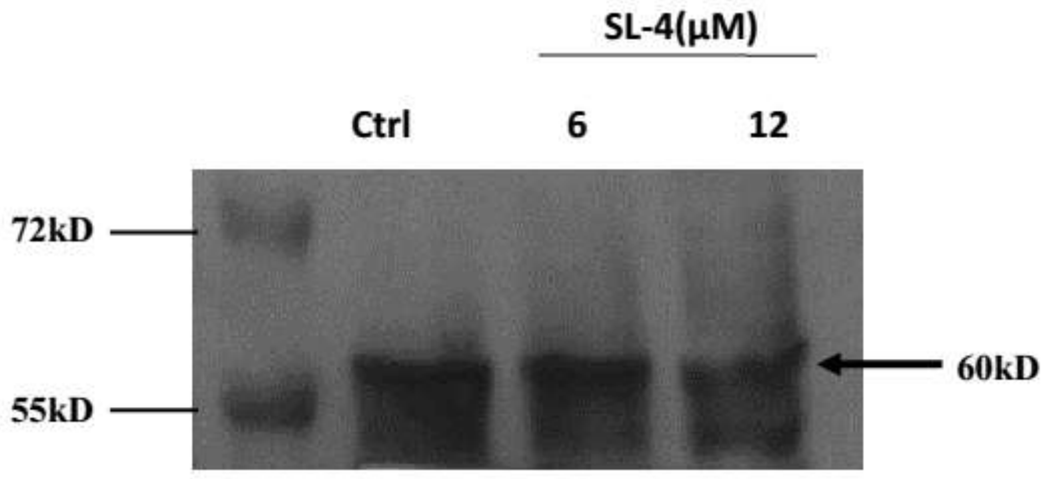


**Supplementary Figure 4-1.  The effects of SL-4 on p-Smad2 in MDA-MB-231 cells.**

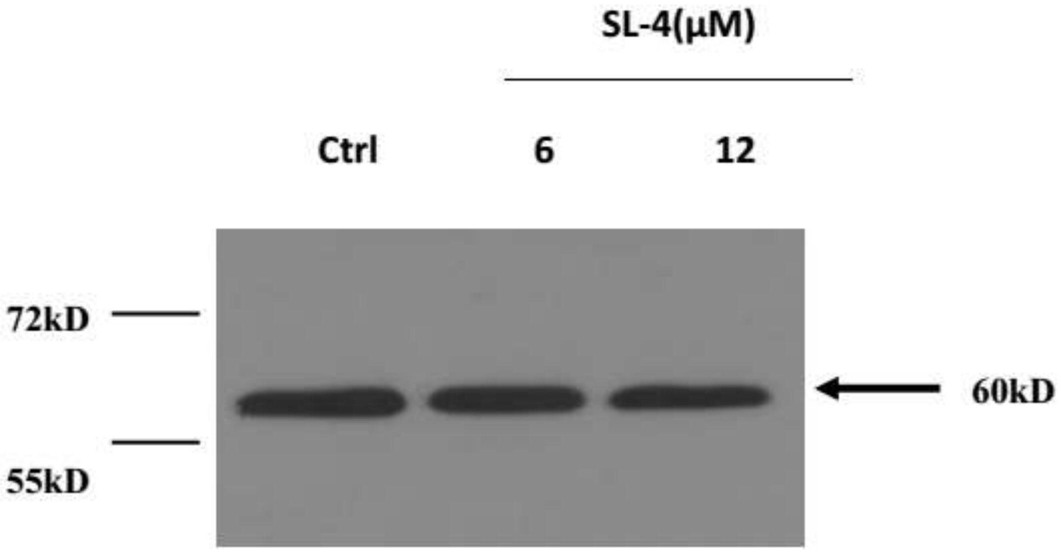


**Supplementary Figure 4-2.  The effects of SL-4 on Smad2 in MDA-MB-231 cells.**

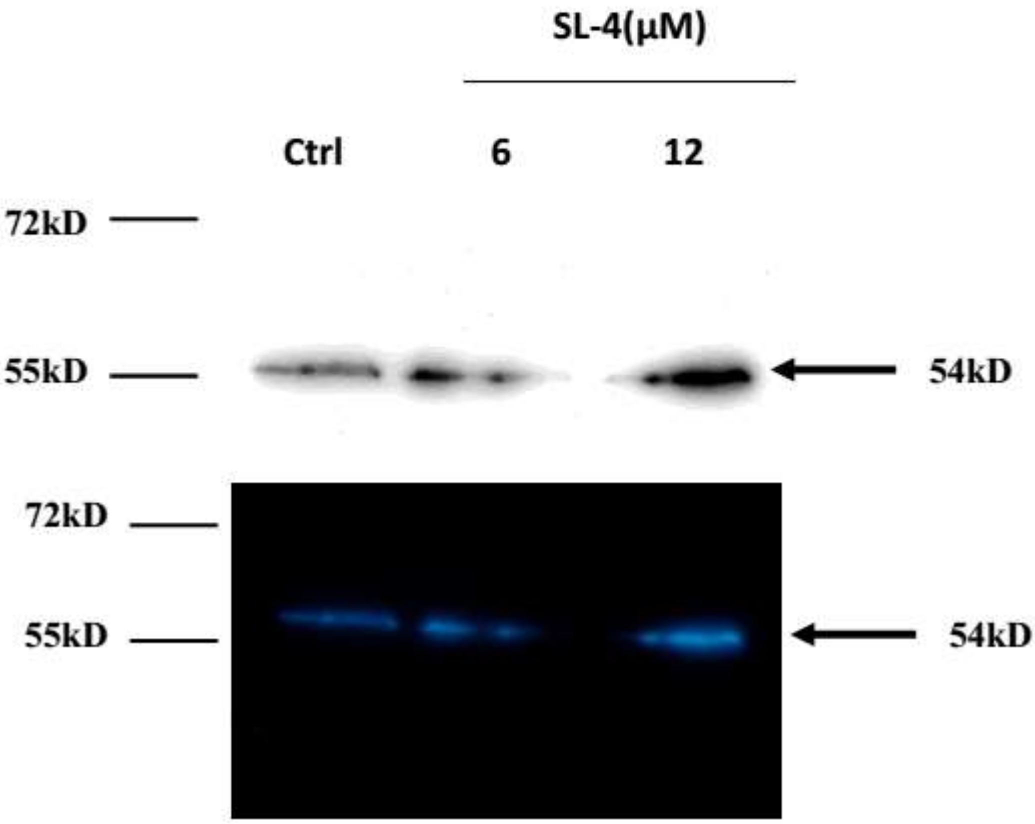


**Supplementary Figure 4-3. The effects of SL-4 on p-Smad3 in MDA-MB-231 cells.**

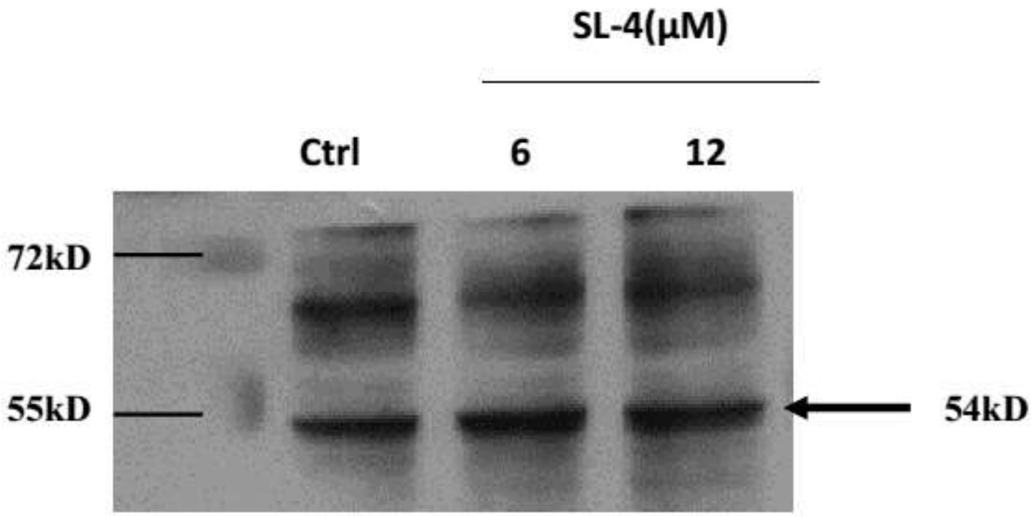


**Supplementary Figure 4-4. The effects of SL-4 on Smad3 in MDA-MB-231 cells.**

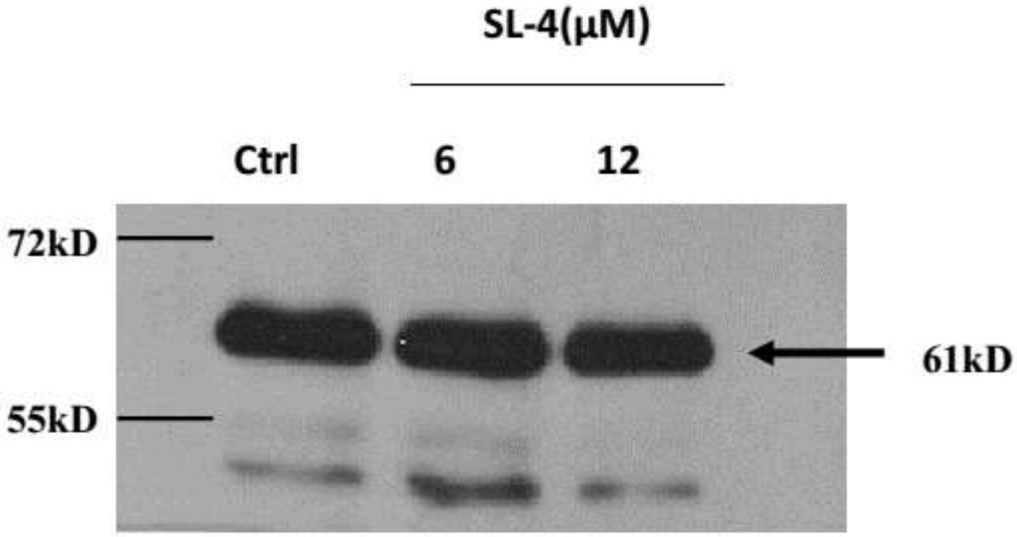


**Supplementary Figure 4-5. The effects of SL-4 on Smad4 in MDA-MB-231 cells.**

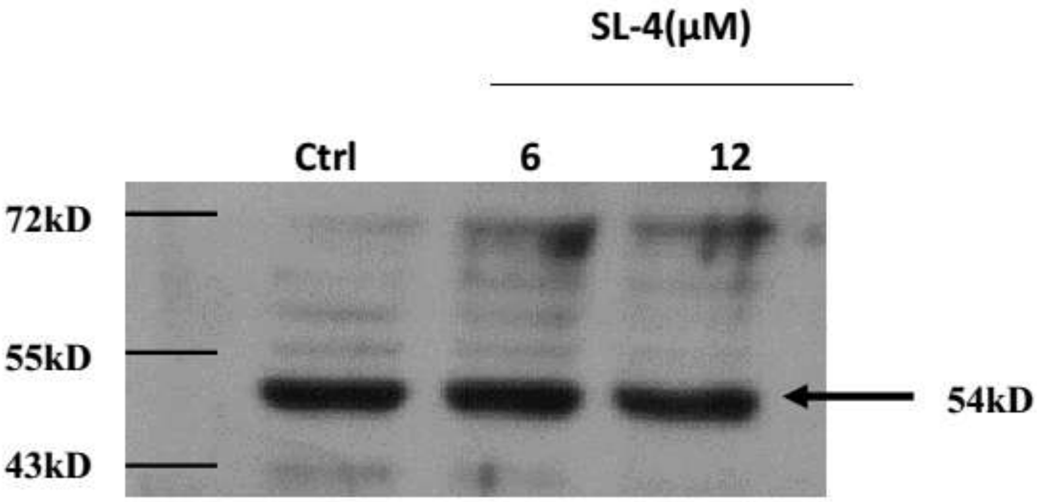


**Supplementary Figure 4-6. The effects of SL-4 on Smad6 in MDA-MB-231 cells.**

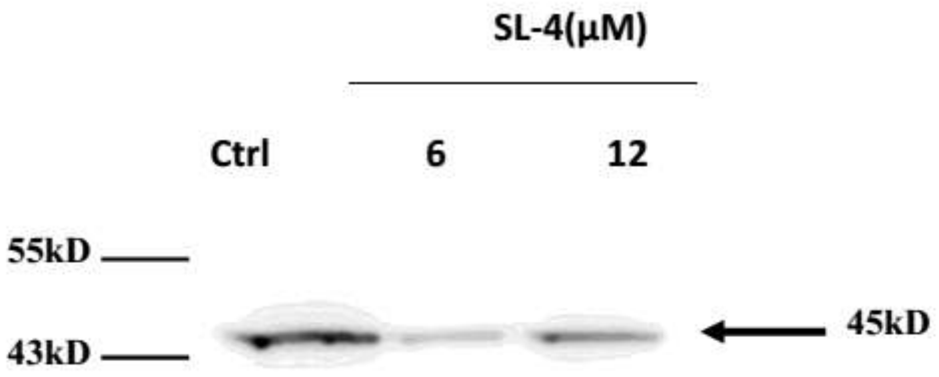


**Supplementary Figure 4-7. The effects of SL-4 on Smad7 in MDA-MB-231 cells.**

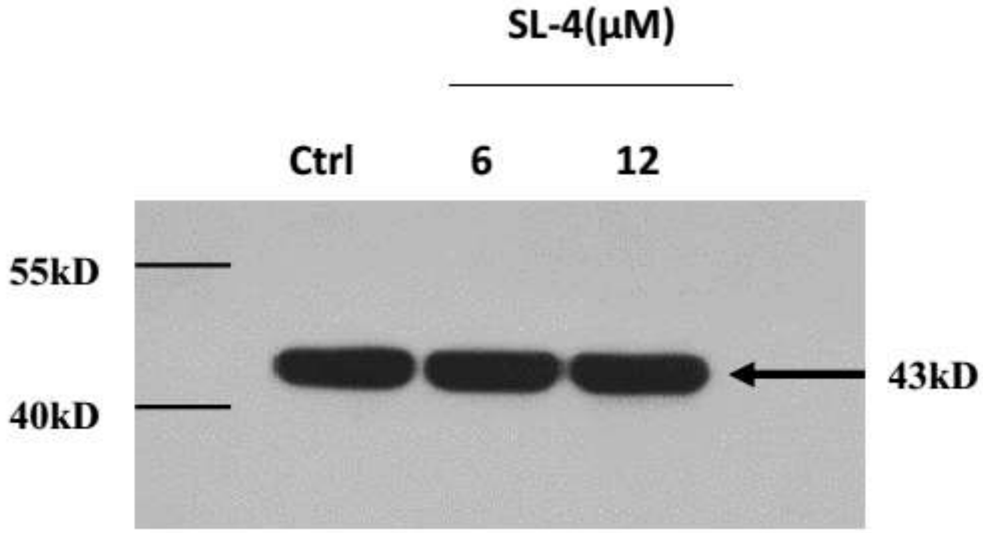


**Supplementary Figure 4-8. Loading control β-actin in MDA-MB-231 cells.**

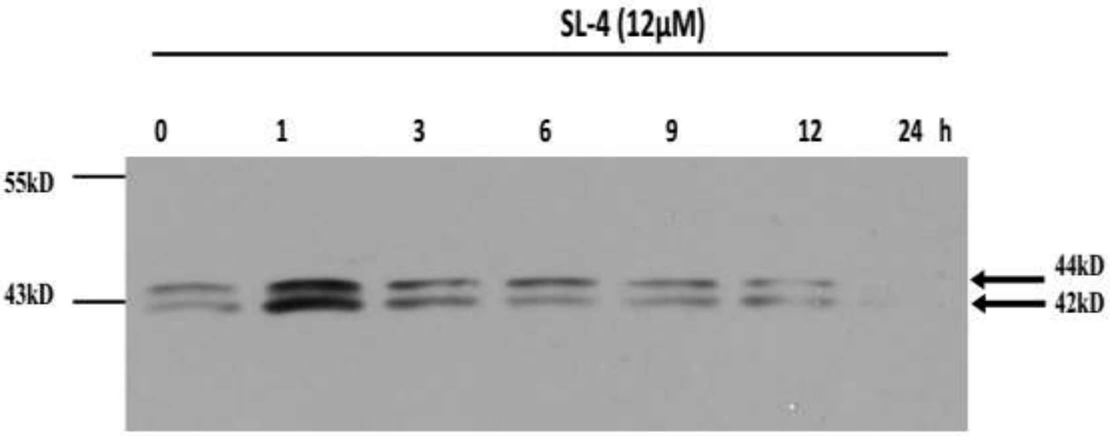


**Supplementary Figure 4-9. The effects of SL-4 on p-ERK in MDA-MB-231 cells.**

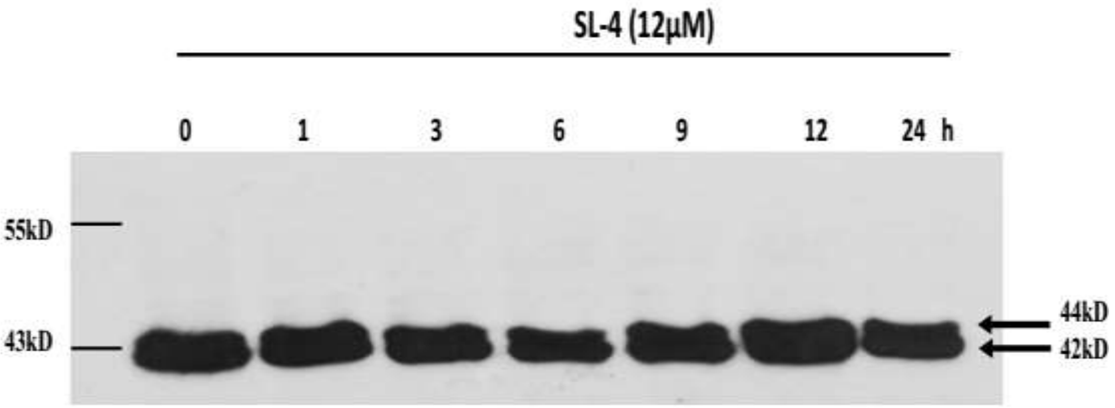


**Supplementary Figure 4-10. The effects of SL-4 on ERK in MDA-MB-231 cells.**

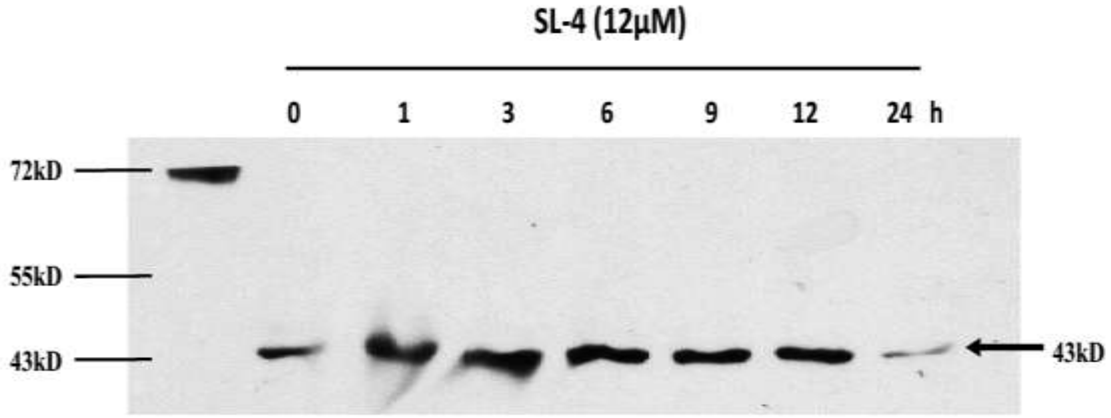


**Supplementary Figure 4-11. The effects of SL-4 on p-p38 in MDA-MB-231 cells.**

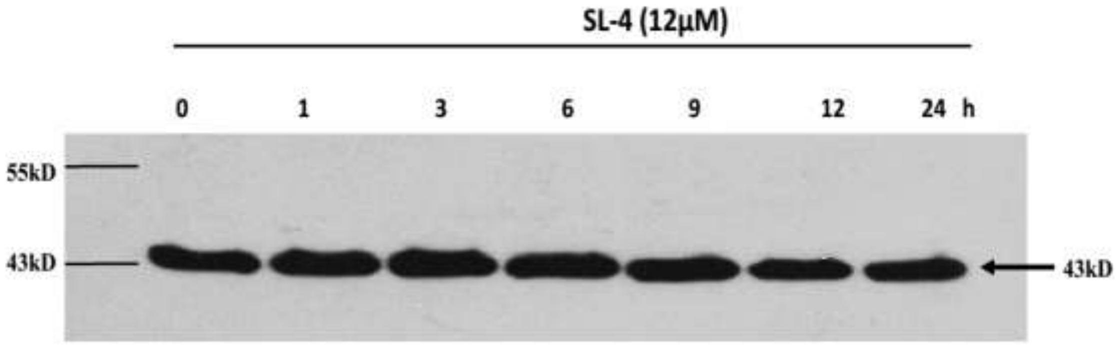


**Supplementary Figure 4-12. The effects of SL-4 on p38 in MDA-MB-231 cells.**

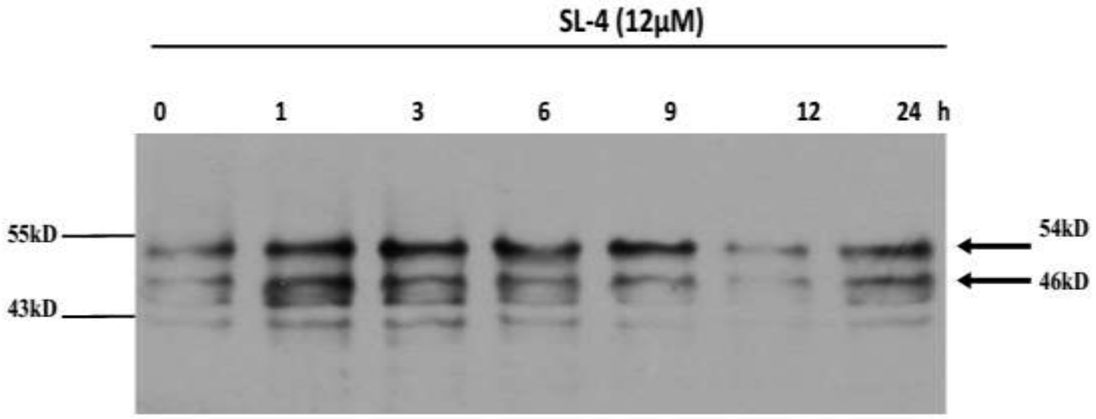


**Supplementary Figure 4-13. The effects of SL-4 on p-JNK in MDA-MB-231 cells.**

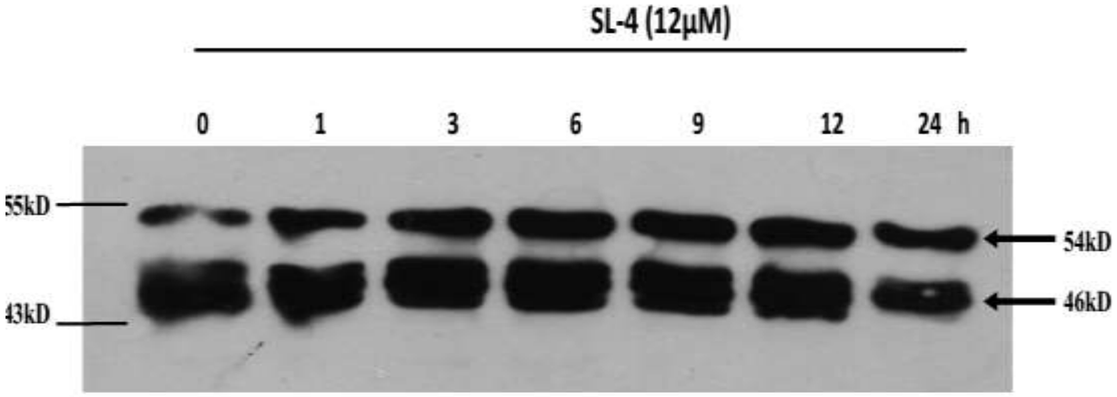


**Supplementary Figure 4-14. The effects of SL-4 on JNK in MDA-MB-231 cells.**

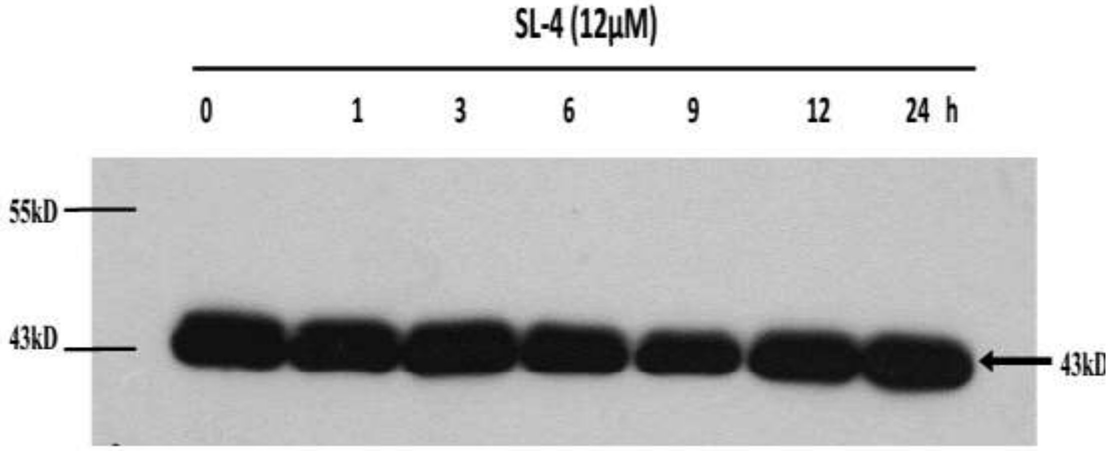


**Supplementary Figure 4-15.** **Loading control β-actin in MDA-MB-231 cells.**

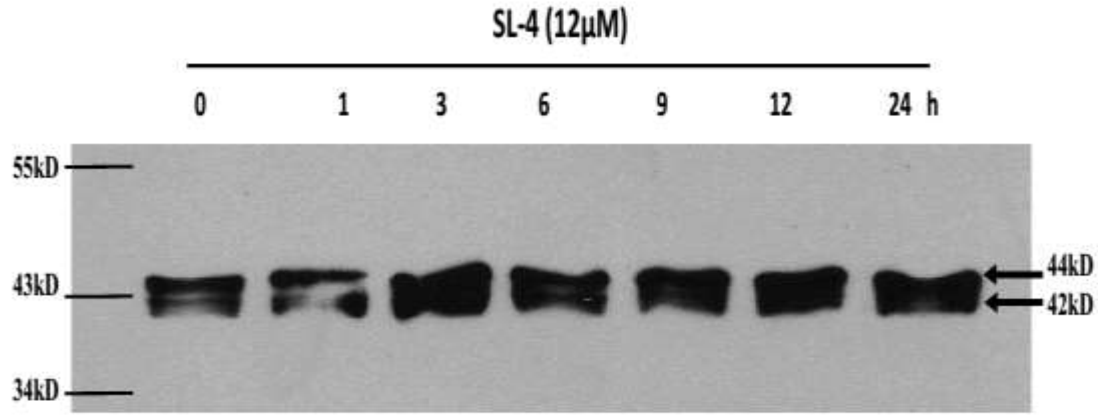


**Supplementary Figure 4-16.** **The effects of SL-4 on p-ERK in MCF-7 cells.**

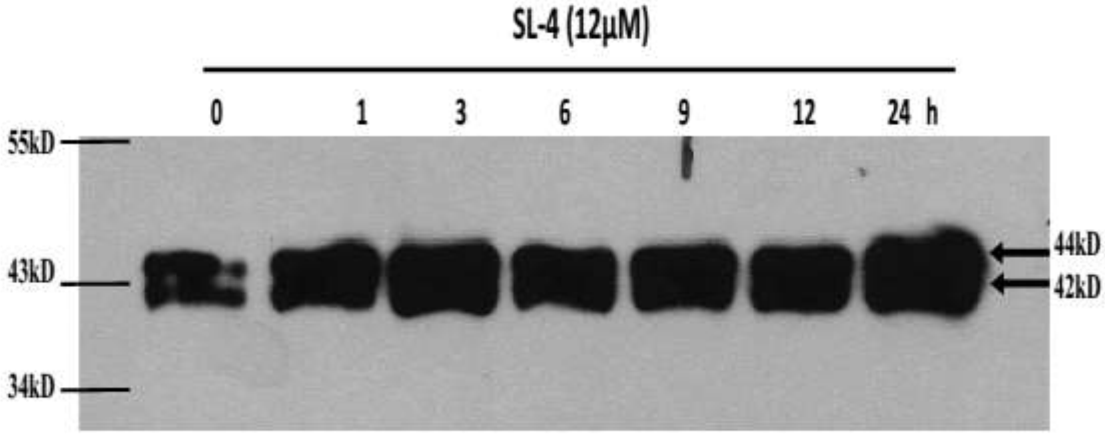


**Supplementary Figure 4-17.** **The effects of SL-4 on ERK in MCF-7 cells.**

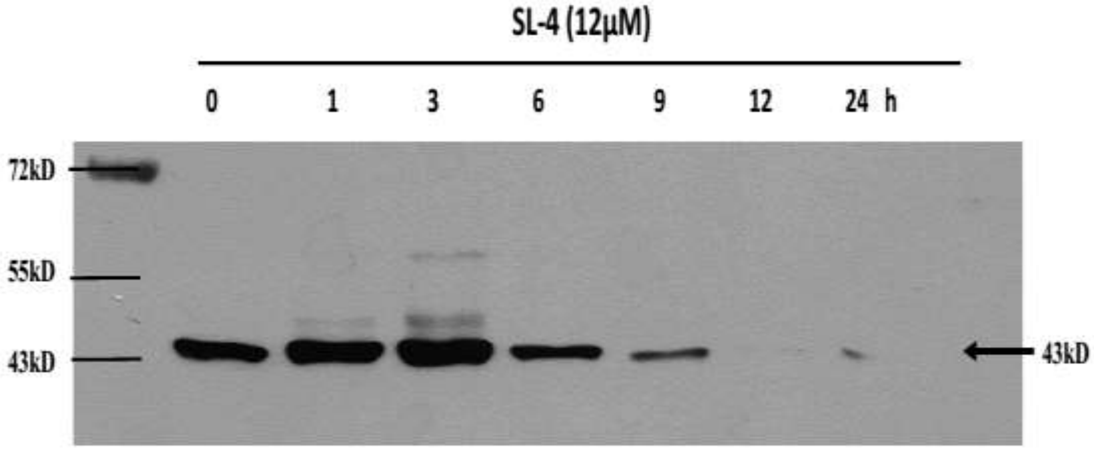


**Supplementary Figure 4-18.** **The effects of SL-4 on p-p38 in MCF-7 cells.**

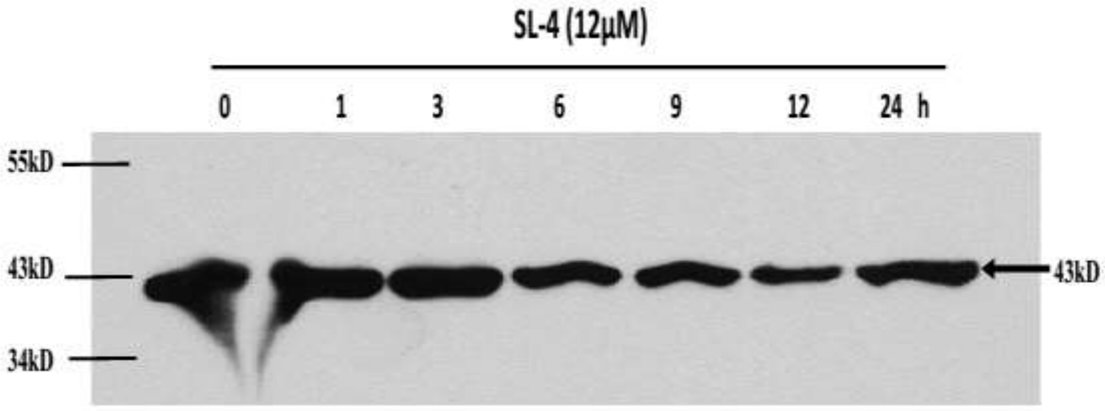


**Supplementary Figure 4-19.** **The effects of SL-4 on p38 in MCF-7 cells.**

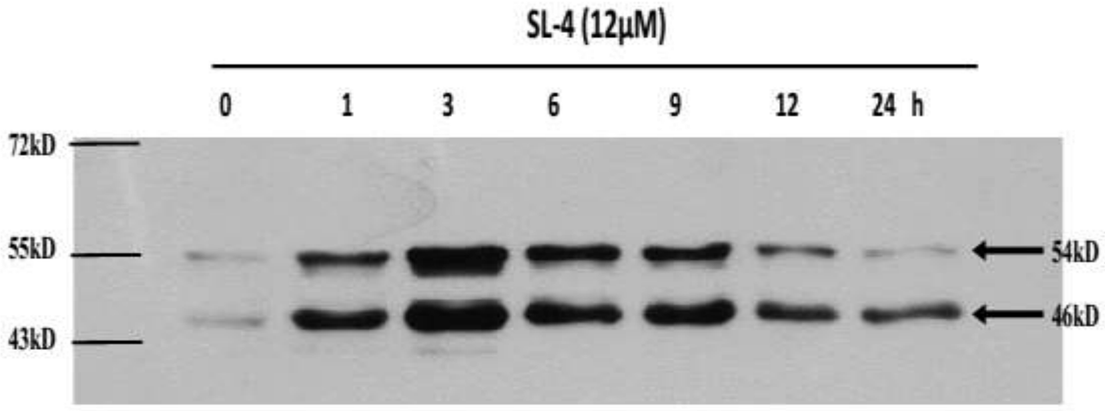


**Supplementary Figure 4-20.** **The effects of SL-4 on p-JNK in MCF-7 cells.**

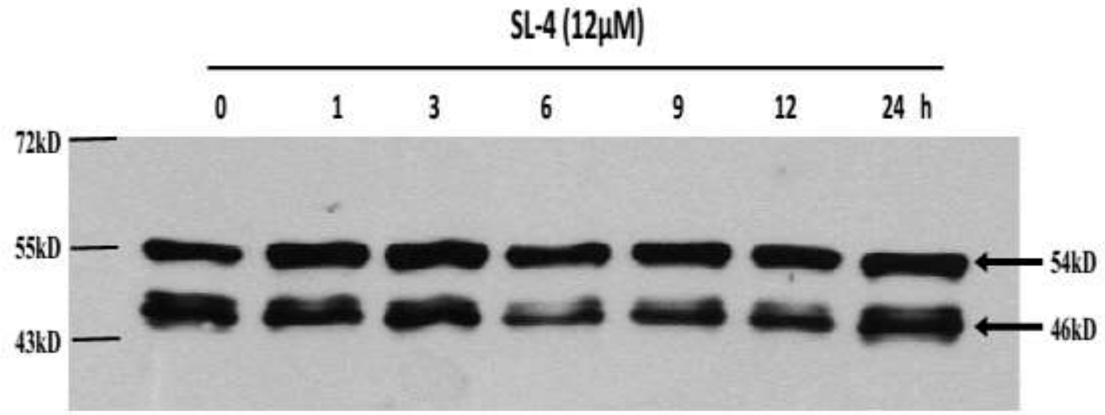


**Supplementary Figure 4-21. The effects of SL-4 on JNK in MCF-7 cells.**

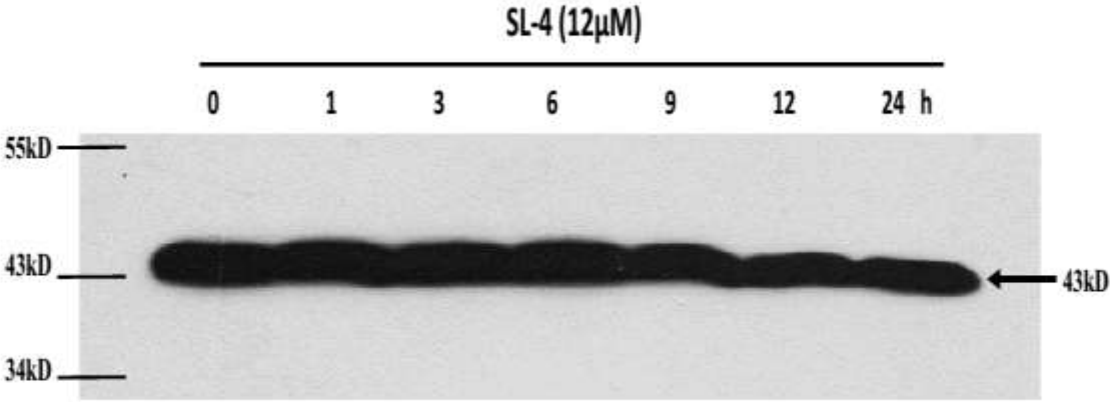


**Supplementary Figure 4-22. Loading control β-actin in MCF-7 cells.**

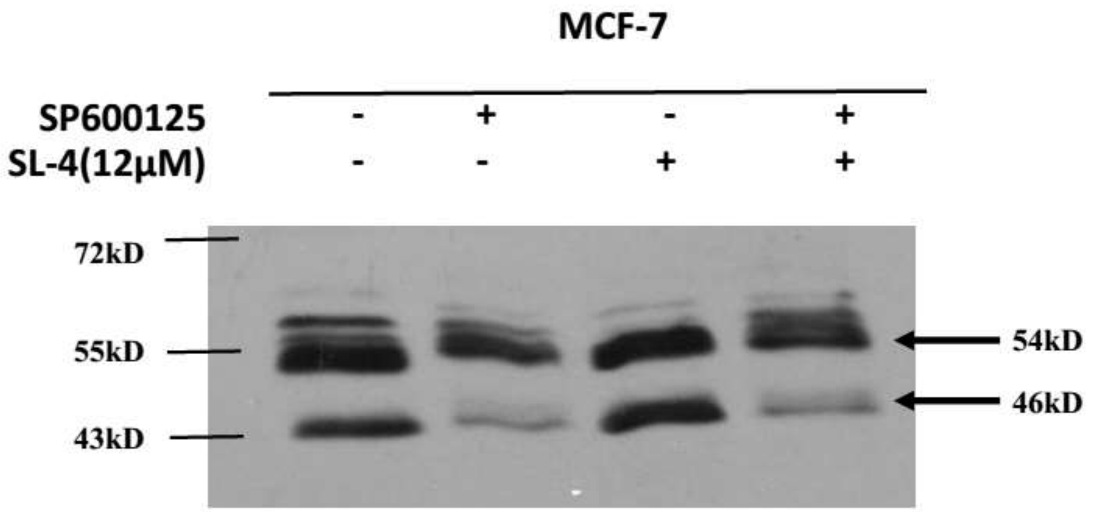


**Supplementary Figure 6-1. The effects of SL-4 or/and SP600125 on p-JNK in MCF-7 cells.**

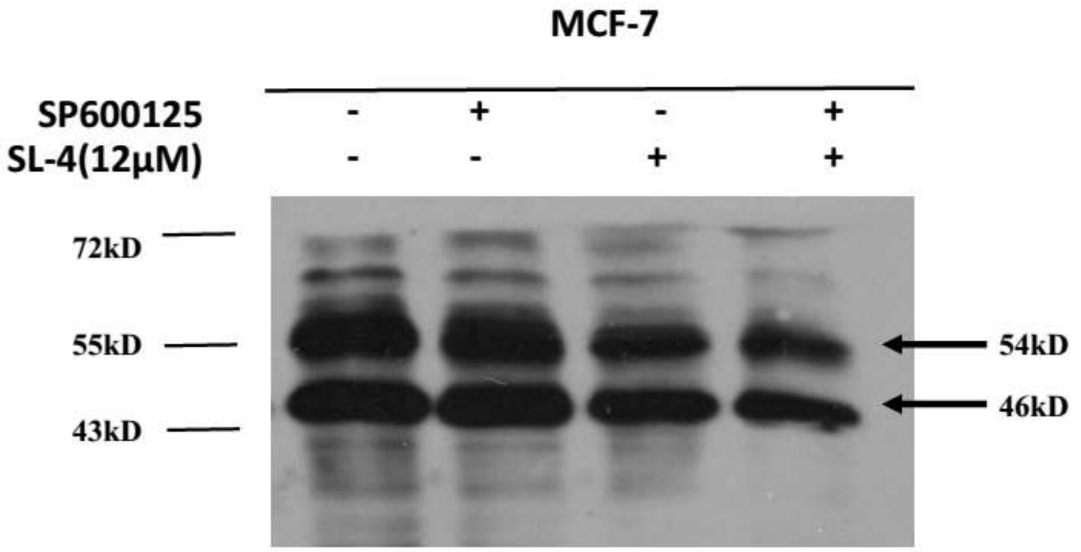


**Supplementary Figure 6-2. The effects of SL-4 or/and SP600125 on JNK in MCF-7 cells.**

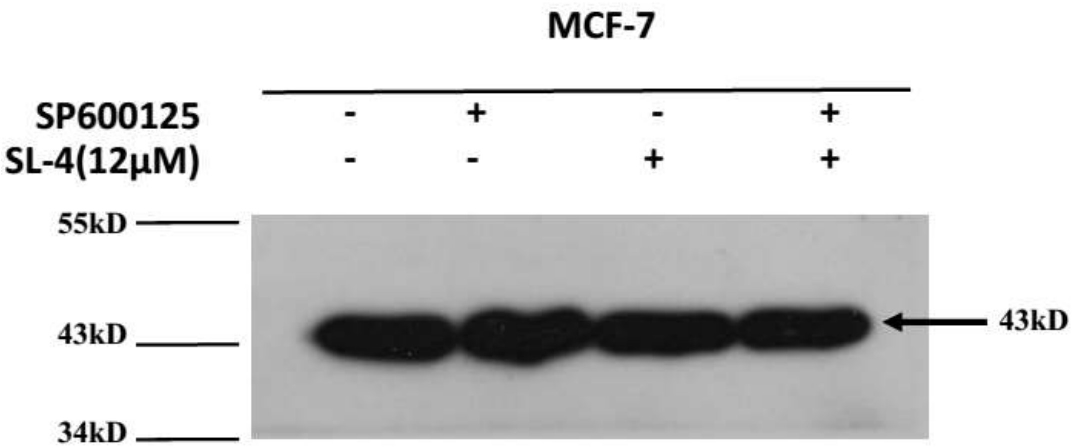


**Supplementary Figure 6-3.** **Loading control β-actin in MCF-7 cells.**

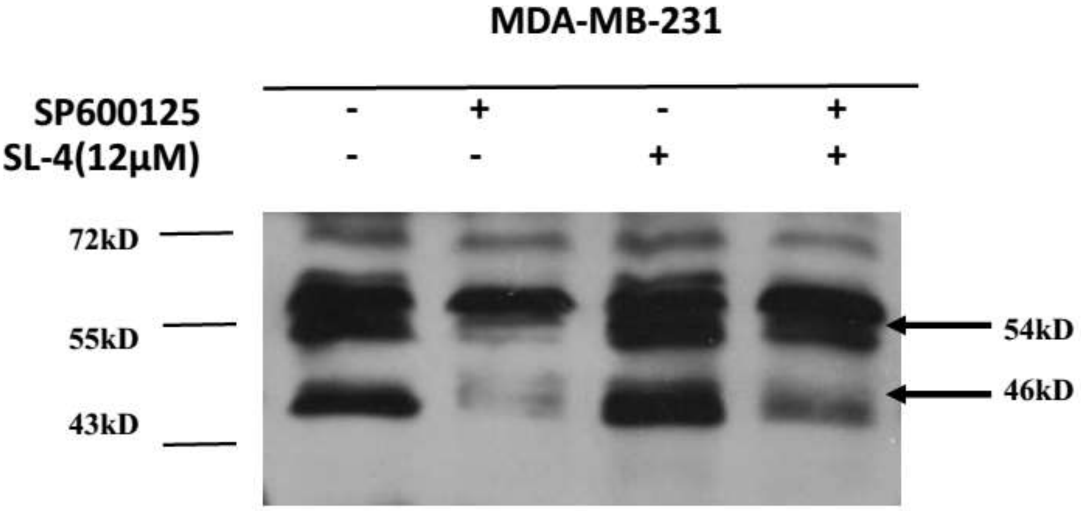


**Supplementary Figure 6-4.** **The effects of SL-4 or/and SP600125 on p-JNK in MDA-MB-231 cells.**

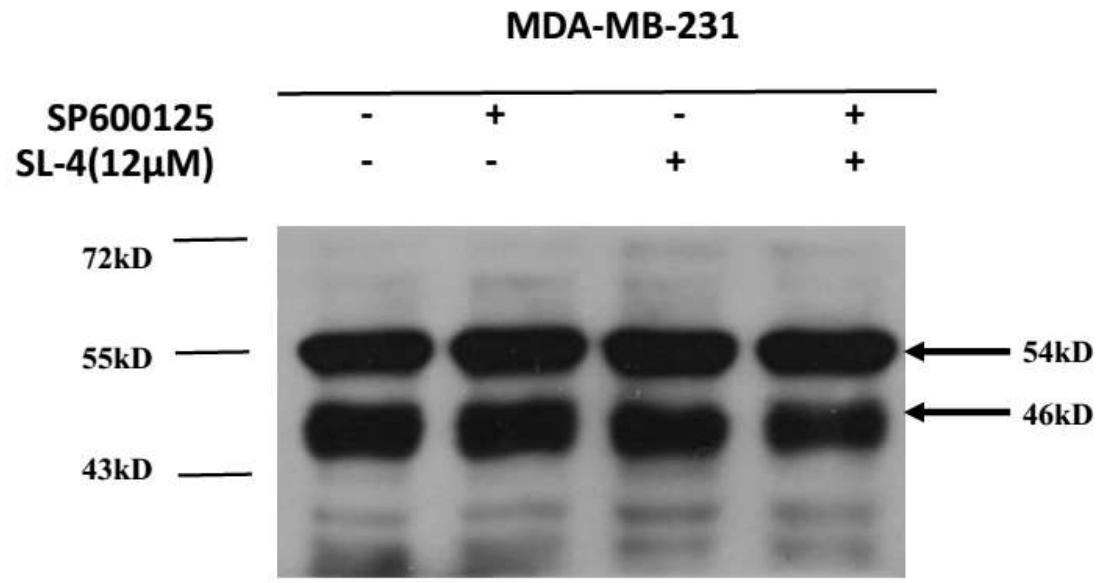


**Supplementary Figure 6-5. The effects of SL-4 or/and SP600125 on JNK in MDA-MB-231 cells.**

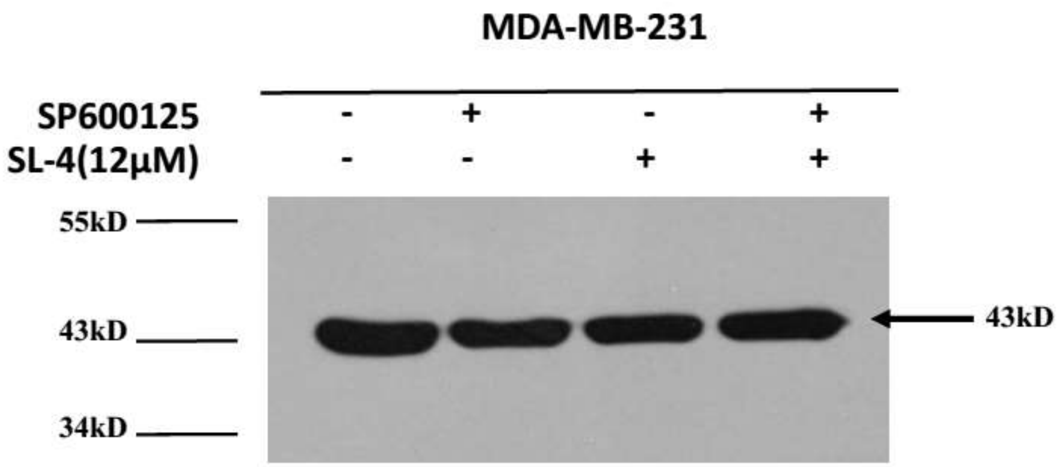


**Supplementary Figure 6-6. Loading control β-actin in MDA-MB-231 cells.**

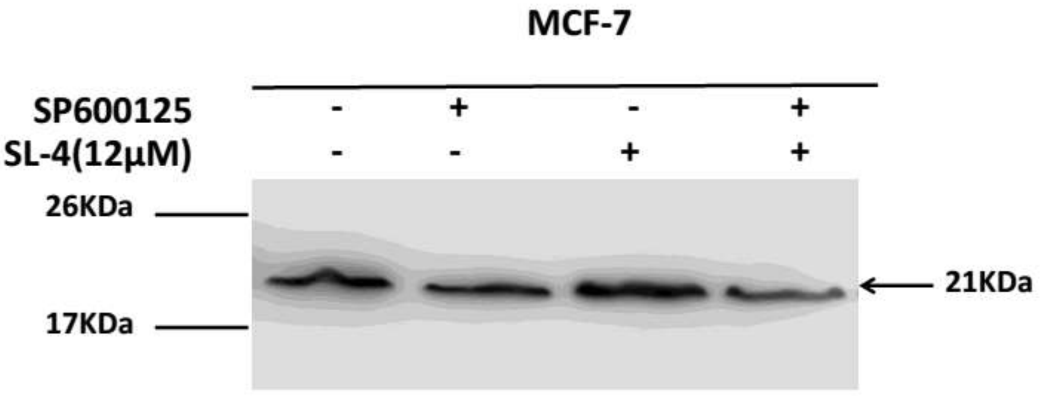


Supplementary Figure 6-7. The effects of SL-4 or/and SP600125 on p21 in MCF-7 cells.

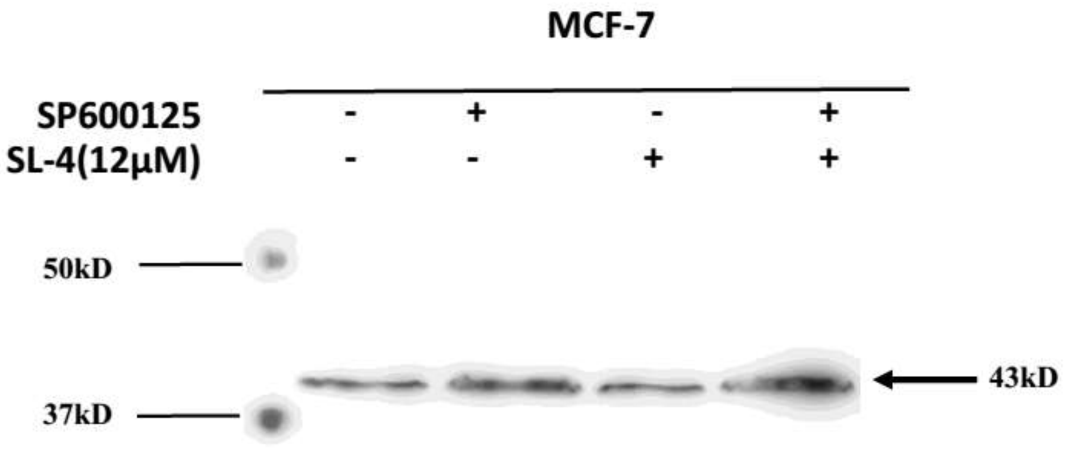


Supplementary Figure 6-8. Loading control β-actin in MCF-7 cells.

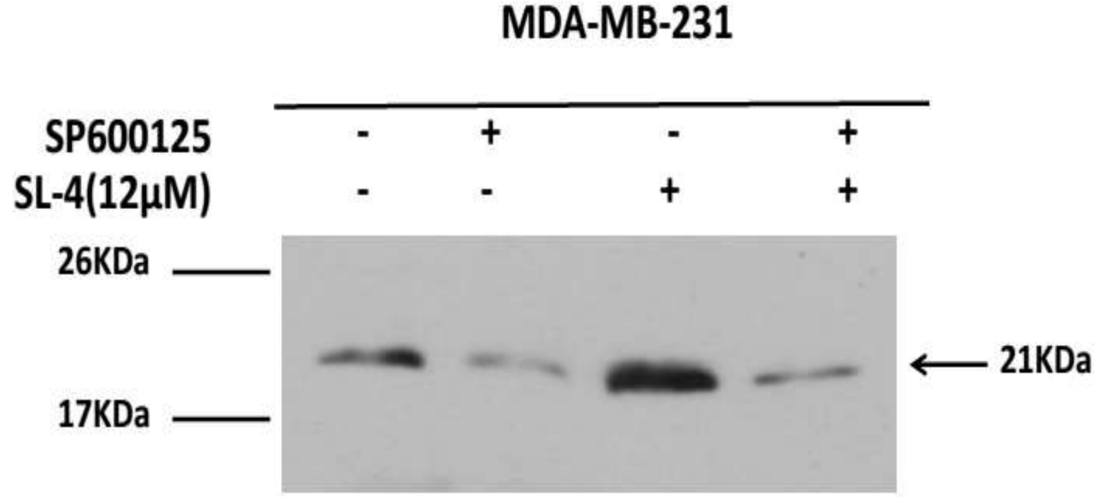


**Supplementary Figure 6-9. The effects of SL-4 or/and SP600125 on p21 in MDA-MB-231 cells.**

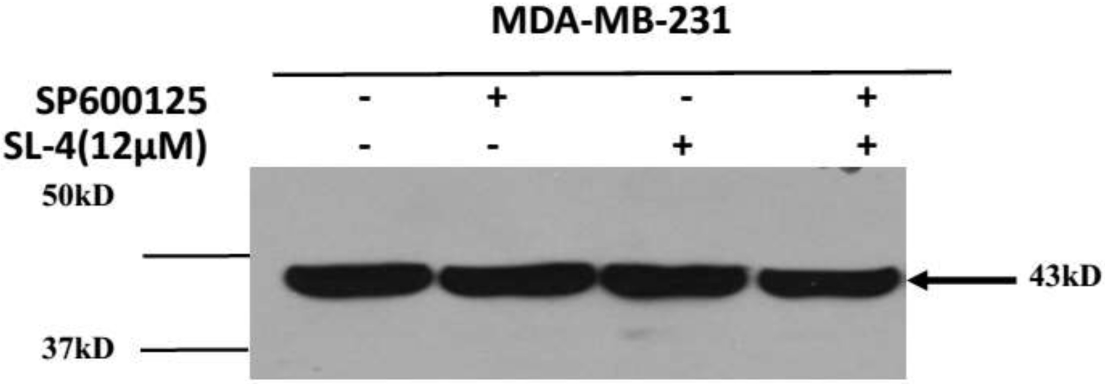


**Supplementary Figure 6-10. Loading control β-actin in MDA-MB-231 cells.**

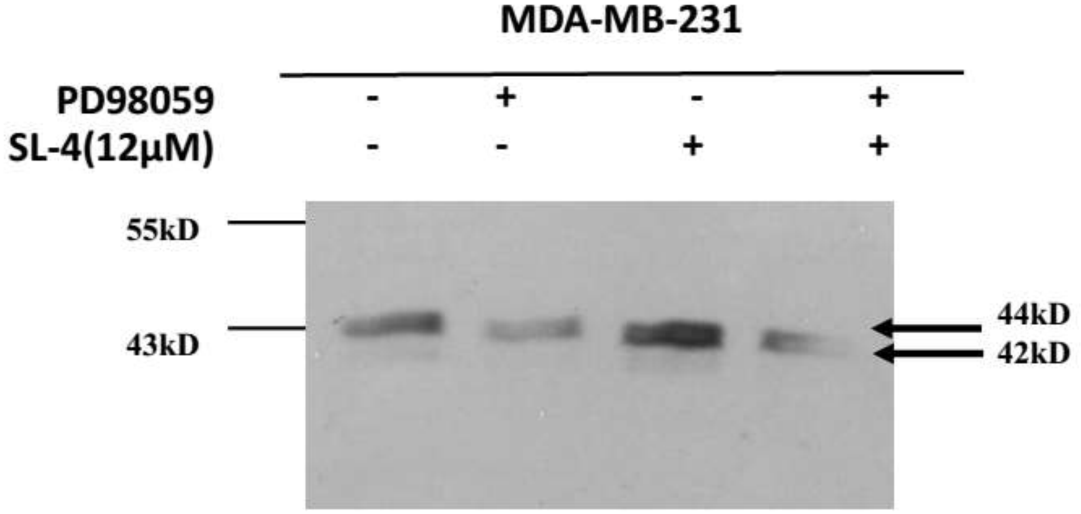


**Supplementary Figure 6-11. The effects of SL-4 or/and PD98059 on p-ERK in MDA-MB-231 cells.**

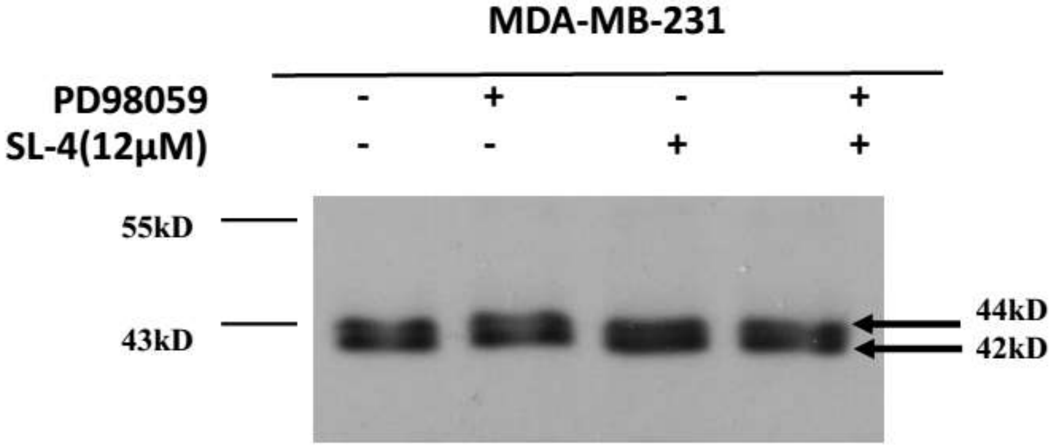


**Supplementary Figure 6-12. The effects of SL-4 or/and PD98059 on ERK in MDA-MB-231 cells.**

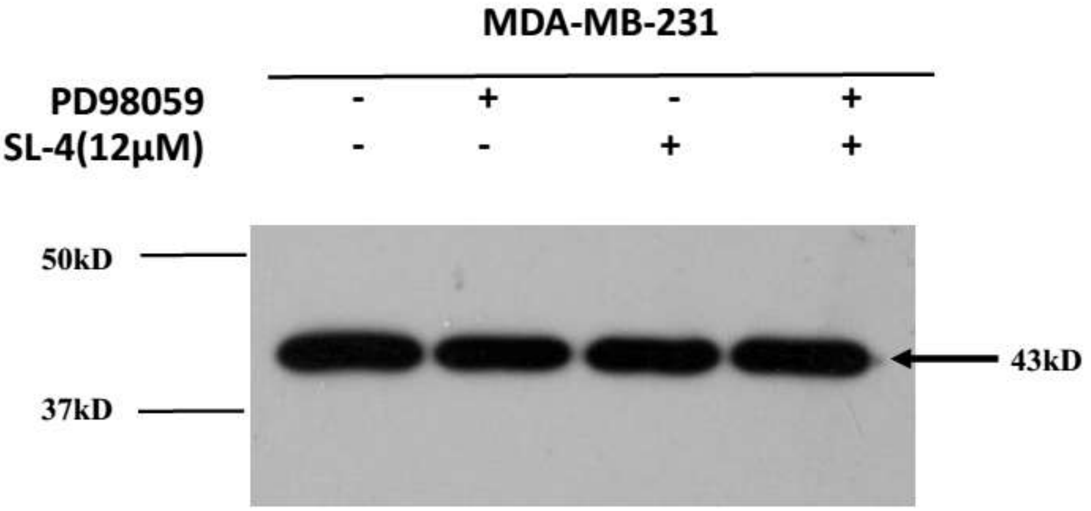


**Supplementary Figure 6-13. Loading control β-actin in MDA-MB-231 cells.**

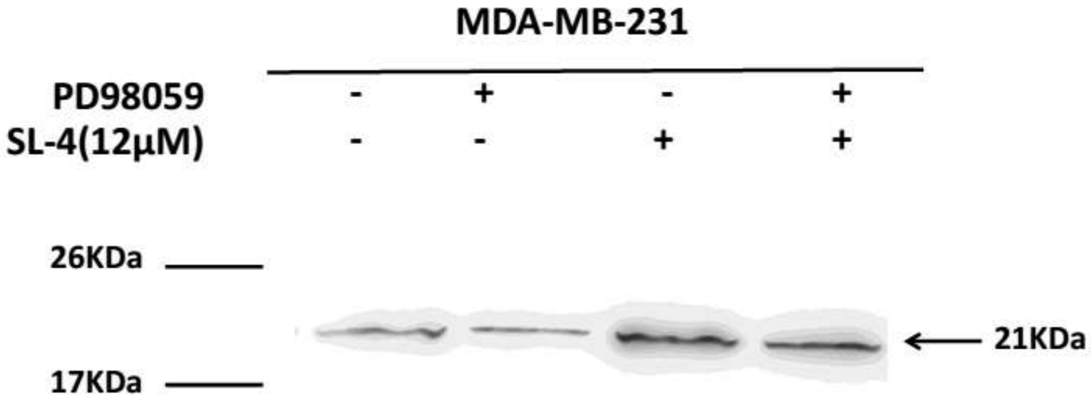


**Supplementary Figure 6-14. The effects of SL-4 or/and PD98059 on p21 in MDA-MB-231 cells.**

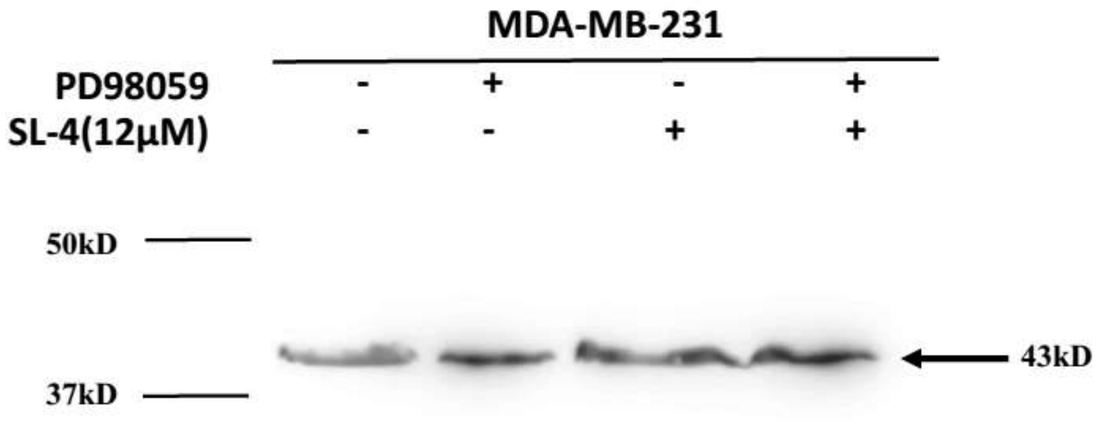


**Supplementary Figure 6-15.** **Loading control β-actin in MDA-MB-231 cells.**

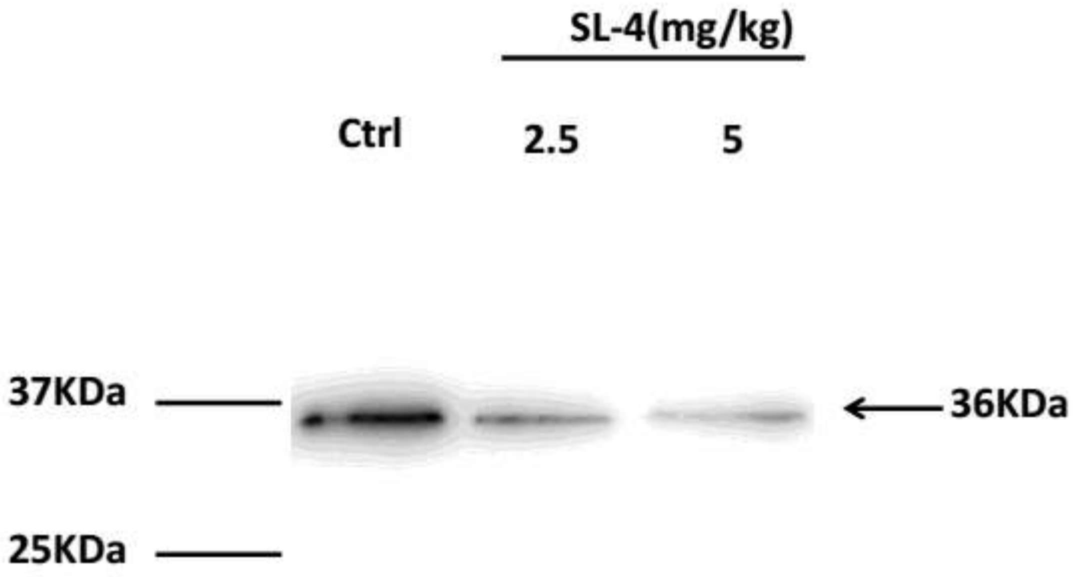


**Supplementary Figure 7-1. The effects of SL-4 on PCNA in MDA-MB-231 xenograft tumor tissues**

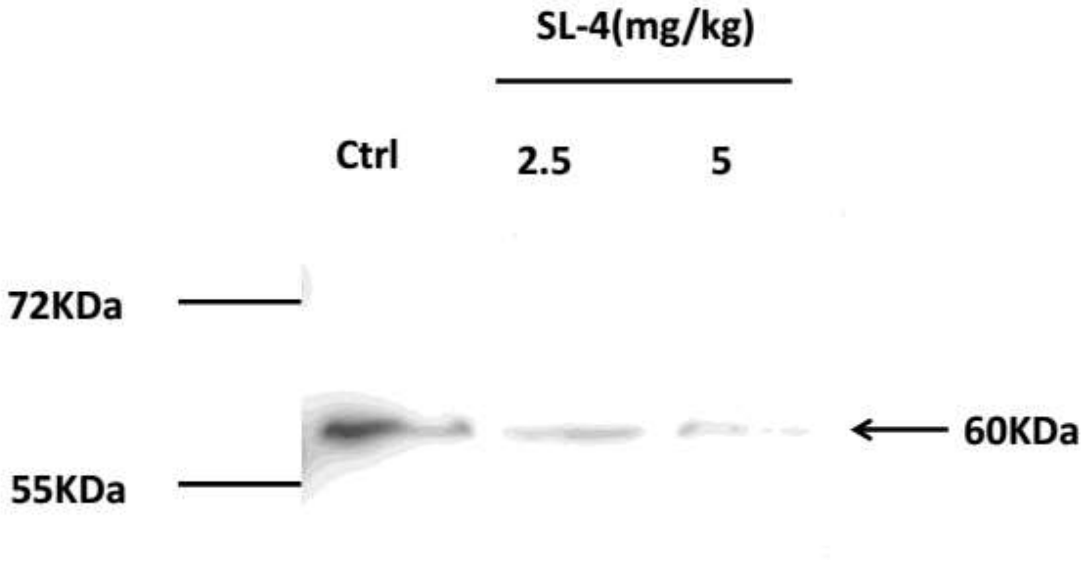


**Supplementary Figure 7-2. The effects of SL-4 on cdc25C in MDA-MB-231 xenograft tumor tissues**

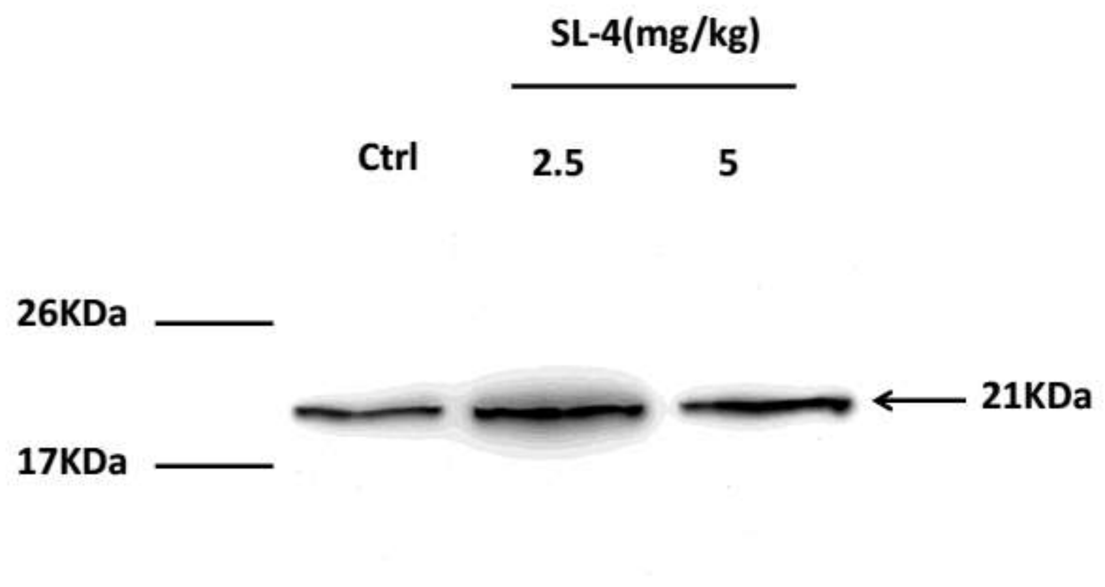


**Supplementary Figure 7-3. The effects of SL-4 on p21 in MDA-MB-231 xenograft tumor tissues**

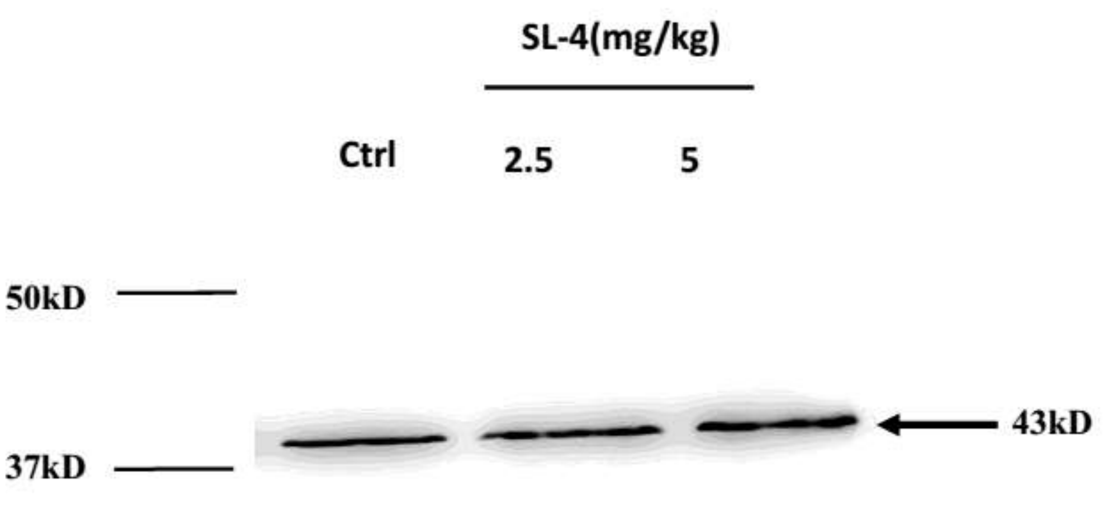


**Supplementary Figure 7-4. Loading control β-actin in MDA-MB-231 xenograft tumor tissues.**
